# Impact of wine bottle and glass sizes on wine consumption at home: a within‐ and between‐ households randomized controlled trial

**DOI:** 10.1111/add.16005

**Published:** 2022-08-02

**Authors:** Eleni Mantzari, Minna Ventsel, Jennifer Ferrar, Mark A. Pilling, Gareth J. Hollands, Theresa M. Marteau

**Affiliations:** ^1^ Behaviour and Health Research Unit University of Cambridge Camvbridge UK; ^2^ School of Psychological Science, Tobacco and Alcohol Research Group University of Bristol Bristol UK; ^3^ EPPI‐Centre, UCL Social Research Institute University College London London UK

**Keywords:** Alcohol, bottle size, consumption, cross‐over, glass size, portion size, randomized trial, RCT, wine

## Abstract

**Background and aims:**

Reducing alcohol consumption across populations would decrease the risk of a range of diseases, including many cancers, cardiovascular disease and Type 2 diabetes. The aim of the current study was to estimate the impact of using smaller bottles (37.5‐ versus 75‐cl) and glasses (290 versus 370 ml) on consuming wine at home.

**Design:**

Randomized controlled trial of households with cross‐over randomization to bottle size and parallel randomization to glass size.

**Setting:**

UK households.

**Participants:**

A total of 260 households consuming at least two 75‐cl bottles of wine each week, recruited from the general population through a research agency. The majority consisted of adults who were white and of higher socio‐economic position.

**Intervention:**

Households were randomized to the order in which they purchased wine in 37.5‐ or 75‐cl bottles, to consume during two 14‐day intervention periods, and further randomized to receive smaller (290 ml) or larger (350 ml) glasses to use during both intervention periods.

**Measurements:**

Volume (ml) of study wine consumed at the end of each 14‐day intervention period, measured using photographs of purchased bottles, weighed on study scales.

**Findings:**

Of the randomized households, 217 of 260 (83%) completed the study as per protocol and were included in the primary analysis. There was weak evidence that smaller bottles reduced consumption: after accounting for pre‐specified covariates, households consumed on average 145.7 ml (3.6%) less wine when drinking from smaller bottles than from larger bottles [95% confidence intervals (CI) = –335.5 to 43. ml; −8.3 to 1.1%; *P* = 0.137; Bayes factor (BF) = 2.00]. The evidence for the effect of smaller glasses was stronger: households consumed on average 253.3 ml (6.5%) less wine when drinking from smaller glasses than from larger glasses (95% CI = –517 to 10 ml; −13.2 to 0.3%; *P* = 0.065; BF = 2.96).

**Conclusions:**

Using smaller glasses to drink wine at home may reduce consumption. Greater uncertainty remains around the possible effect of drinking from smaller bottles.

## INTRODUCTION

Alcohol consumption is a major contributor to premature death and disease globally [1]. Reducing alcohol consumption at the population level would decrease the risk of a range of non‐communicable diseases, including some cancers, cardiovascular disease and Type 2 diabetes [2]. Interventions that target aspects of the physical environments that cue unhealthy behaviour, such as product affordability, availability and size, have significant potential to have scalable impacts at a population level, including on reducing harmful alcohol consumption [3, 4, 5, 6, 7, 8].

Wine is the most commonly drunk alcoholic beverage in Europe, including the United Kingdom. Most wine is consumed in homes rather than in bars, restaurants or pubs [9, 10]. Although a bottle containing 75 cl is now widely accepted as the standard size for wine [11], more recently smaller bottles, in particular those containing 37.5 cl, have become more widely available in many countries, including the United Kingdom [12, 13, 14, 15]. Smaller portions and packages decrease the consumption of food and non‐alcoholic drinks [16]. In terms of alcoholic drinks, altering the size of containers in which wine is packaged, sold and served has the potential to reduce consumption. Specifically, smaller wine bottles may reduce both the amount consumed and the rate of consumption, as found in a recent randomized cross‐over trial in which households consumed 4.5% less wine at home from 50‐cl bottles than from 75‐cl bottles [17]. The impact of more widely available 37.5‐cl bottles is unknown, however. The results of a feasibility and acceptability study, comparing consumers’ responses to 75‐ and 37.5‐cl bottles, highlighted the possibility that the latter could increase rather than decrease consumption [18]. Although this study was not designed to estimate differences in consumption with the different bottle sizes, it suggests that the amount held in 37.5‐cl bottles could, on occasion, be considered too small, especially given that 75‐cl bottles have become the standard size for wine internationally, potentially leading to multiple bottles being consumed per drinking occasion. Smaller bottles could also increase consumption by reducing barriers to consumption that are present for larger sizes [19], such as inhibitions regarding opening larger bottles to avoid over‐consumption or wastage posed by the availability of wine in larger bottles.

The size of glasses in which alcohol is served can also influence the amount consumed, with larger glasses increasing the volume of wine sold, and therefore assumed to have been consumed, in restaurants by approximately 7.3% [20]. The size of wine glasses used with different‐sized bottles may enhance or diminish any effect of bottle size. A recent laboratory study in which participants were asked to pour wine into three differently sized glasses (small, medium, large) from two differently sized bottles (50 and 75 cl) found that less wine was poured into smaller glasses, but this was unaffected by bottle size [21]. The impact of glass size singly and in combination with different bottle sizes when multiple servings are allowed is unknown. Most importantly, there is no evidence from in‐home settings—where most alcohol is consumed—in the countries that consume most wine.

The primary aim of the current study was to addresses this evidence gap by estimating the impact on the volume of wine consumed at home from using different bottle sizes in combination with different glass sizes. It was hypothesized that less wine would be consumed at home using:

(a) smaller versus larger bottles;
(b) smaller versus larger glasses; and
(c) smaller bottles with smaller wine glasses versus larger wine bottles with larger wine glasses.

Given that consumption rate is associated with amount consumed [22, 23, 24], the secondary aim of the study was to assess the impact of bottle and glass sizes on the rate of wine consumed in households. This was defined as the mean number of days taken to consume each 1.5 l of wine for each bottle–glass size combination.

## METHODS

The study was approved by the University of Cambridge Psychology Research Ethics Committee (reference no: PRE.2020.098). The study protocol was pre‐registered (ISRCTN: ISRCTN16597253, https://www.isrctn.com/ISRCTN83786867; Open Science Framework: registration: https://osf.io/efksz/; protocol: https://osf.io/9u684/). The statistical analysis plan was pre‐specified and uploaded to the Open Science Framework (OSF) prior to the start of data analysis: https://osf.io/gvh2a/.

### Study design

This study used a randomized controlled trial of households with cross‐over randomization to bottle size and parallel randomization to glass size. Bottle size conditions were separated by a ‘usual behaviour’ washout period. As it was not feasible to reliably change the size of wine glasses used within households, parallel rather than cross‐over randomization was chosen for glass size.

### Participants

Data were collected from 260 households—defined as people living together—in the United Kingdom between November 2020 and August 2021. Eligible households were of any size or composition in which adult members: together drank a minimum of 2 × 75 cl bottles a week; were in possession of a device, such as a smartphone, from which to take and send photographs of wine consumed; did not take medications for which there was a recommendation against alcohol consumption; did not have a serious mental illness, history of alcoholism, or of becoming ill enough to require hospitalization after alcohol consumption; were not pregnant or planning to become pregnant during the study period.

All potentially eligible households were recruited via a research agency (Roots Research https://rootsresearch.co.uk/).

#### Sample size

The sample size calculation was informed by a previous cross‐over RCT, which found a reduction in consumption of 191.1 ml [standard error (SE) = 76.5, *t* = 2.49] when drinking wine from 50‐cl bottles compared to 75‐cl bottles [17], an estimated effect size of *d* = 0.196. To detect such an effect of bottle size with 80% power at the 5% significance level, using a 2 × 2 within‐subjects design (i.e. allocation order by period, ignoring glass size) a total sample size of 206 was required. This sample size also gives 80% power at the 5% significance level to detect a glass size effect of 0.39 or larger, or an interaction effect size between bottle size and glass size of 0.22 or larger.

### Randomization

Households were randomized to the order in which they were instructed to purchase wine in the two different bottle sizes. They were also randomized to drink wine using either smaller or larger wine glasses. Both randomizations occurred via Qualtrics during completion of a on‐line baseline questionnaire using the platform’s randomization software. The first occurred after the screening questions, and allocated eligible households to small or large glasses. The second occurred after the demographic questions and households had been allocated to small or large bottles. Blocked randomization was used to ensure that approximately equal numbers of households were randomized to receive smaller or larger wine glasses and to the bottle size to purchase first, using the ‘evenly presented elements’ function.

### Interventions

#### Bottle size

**FIGURE 1:**
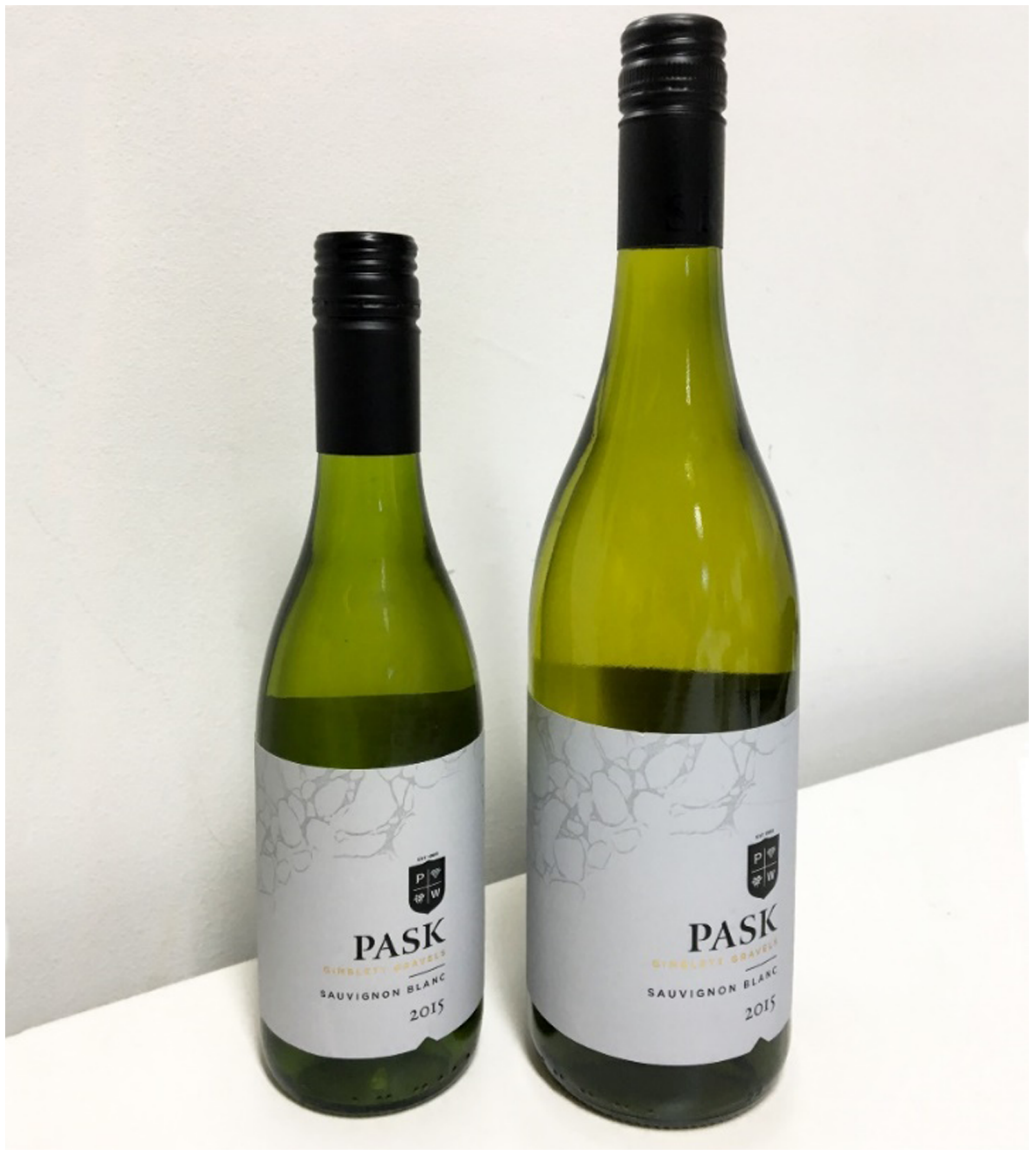
Example of wine bottles used in the study (left: 37.5‐cl bottle; right: 75‐cl bottle) [Color figure can be viewed at wileyonlinelibrary.com]

The intervention comprised purchasing a given quantity of wine—based on the self‐reported volume consumed per household at baseline—in bottles of one of two different sizes, in an order determined by randomization (Figure 1): (i) 37.5 cl and (ii) 75 cl. Study wines for each variety and producer were available in both bottle sizes (see Supporting information, Appendix S2 for the study wine lists).

Each intervention period lasted 2 weeks (14 days). There was an intervening ‘washout’ period lasting between 0 to 3 weeks—with a longer duration permitted in some circumstances—to allow households to finish the wine ordered during the first intervention period. Households were only able to start the second intervention period once all the wine had been consumed from the first period.

#### Glass size

**FIGURE 2:**
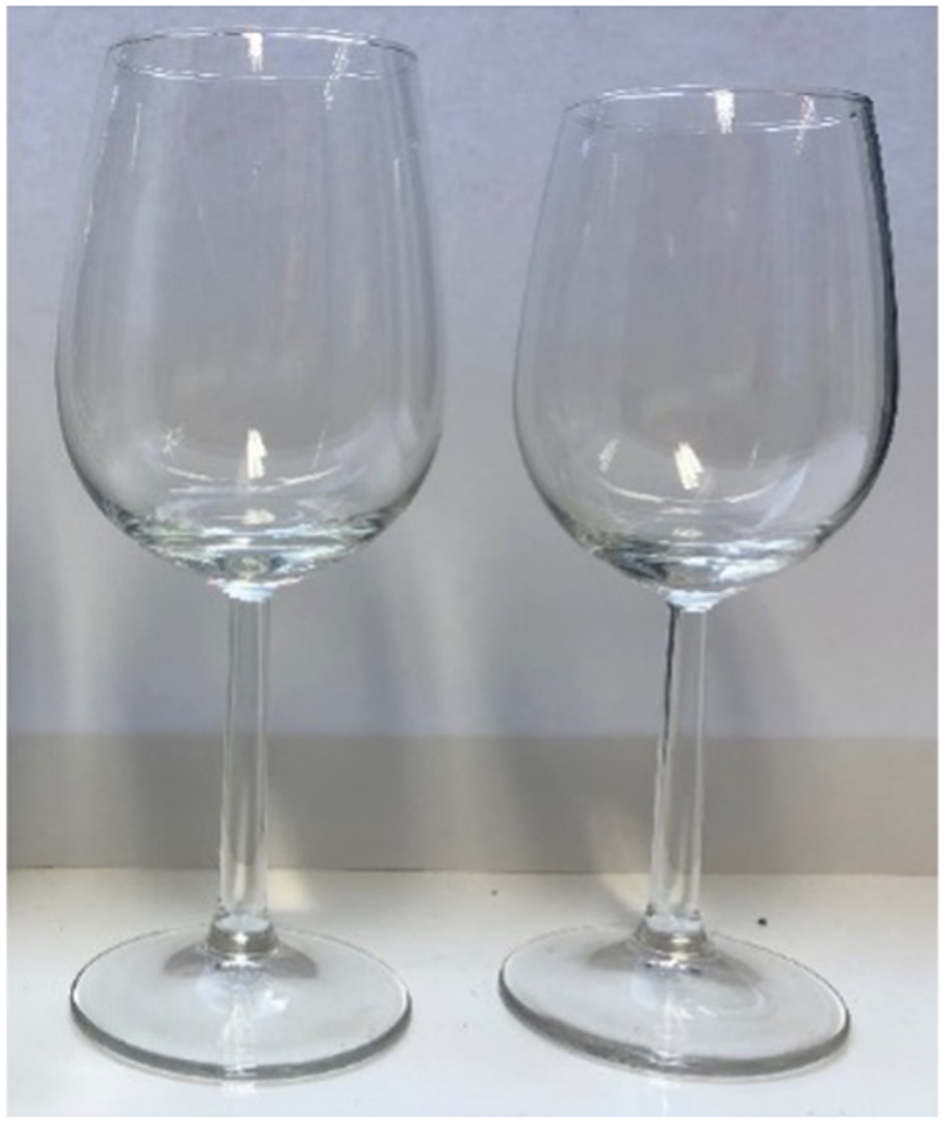
Wine glasses used in study (left: 350 ml; right: 290 ml) [Color figure can be viewed at wileyonlinelibrary.com]

The intervention comprised drinking the study wine from one of two wine glass sizes, allocated through randomization (Figure 2): (i) 290‐ml capacity and (ii) 350‐ml capacity. Both glass sizes were of the same design (Royal Leerdam Bouquet).

Both bottle and glass size interventions are classified as size × product intervention in the typology of interventions in proximal physical microenvironments (TIPPME) [25].

### Procedure

See Supporting information, Appendix S1 for full details of information provided to participants.

Representatives of potentially eligible households, i.e. individuals recruited from each household to provide the data, were sent detailed information about the study, including a link to an instructional video explaining the various stages of the study. They were also directed to an on‐line baseline questionnaire conducted on Qualtrics to assess their eligibility. All household representatives provided written consent before study enrolment. In an attempt to mask the true aim of the study, participants were told the study was exploring the impact of bottle size and glass on the experience of wine drinking, including taste and pleasure, rather than on the quantity of wine consumed. In reality, the nature of the study was not exploratory and this was not its true aim; this was simply a cover story designed to take attention away from the true focus of measuring quantities of wine.

Households were randomized to their first bottle size condition, i.e. to first purchase wine in either 75‐ or 37.5‐cl bottles. Representatives were asked to select the wines they would like to receive for the first intervention period. They were then redirected to the retailer’s website with instructions to purchase their preferred wine in the allocated bottle size, in quantities based on 3 weeks’ typical self‐reported consumption. The amounts were fixed during both intervention periods. Representatives sent their order confirmation to the research team.

Upon receipt of their order confirmation, the research team sent participating households a set of written instructions and a link to a video explaining the study procedures, including how to take days 7 and 14 photographs. They were also sent sticky labels to attach to the study wine bottles to record the following details: the date each bottle was opened and finished; the number of household members and guests who drank from the bottle together with estimated volumes drunk by guests; and the volume of any non‐study wine consumed at home. Depending on their allocated glass size condition, households were also sent a set of wine glasses, with the exact number depending on the number of wine drinkers within that household. Finally, households were sent a pair of study scales (Tower Kitchen Scales) with batteries.

Receipt of all the study items, including the wine bottles, marked the beginning of the first intervention period. Household representatives sent photographs of all bottles (with applied labels) weighed on study scales to the researchers on days 7 and 14 of each intervention period. To assess fidelity to glass size, households were also requested to send photographs of the glasses they had used on days 7 and 14 of the intervention periods. Each household representative received an e‐mail reminder on the day their photographs were due, as well as a follow‐up e‐mail the day after the due date if the photographs had not already been received. Photographs were checked upon receipt and any queries followed‐up with participants. Once photographs were approved, participants were e‐mailed and asked to complete an on‐line questionnaire to assess out‐of‐home wine consumption, and any mitigating factors affecting in‐home consumption.

At the end of the first 14‐day intervention period, if required the households completed a ‘usual behaviour’ washout period to allow any remaining wine to be finished. During this period, there were no constraints on bottle size or glass size or types of wine that households could drink. Once all the wine was consumed or was close to being consumed, household representatives were directed to a second on‐line questionnaire informing them of the bottle size and quantity to order for their next intervention period. Households were asked to order the same volume as during the first intervention period, but in the new bottle size. Receipt of the second wine order marked the beginning of the second intervention period, during which the first intervention period procedures were followed.

At the end of the study, household representatives were fully debriefed on the study aims of and received £242 in total for completing the study in full. Participants were not paid for wine purchases made during the study.

### Outcome measures

#### Primary outcome

Volume of study wine consumed (in millilitres: ml) during each 2‐week intervention period for each bottle–glass size combination, assessed through returned photographs of all study wine bottles purchased.

Volumes consumed from opened bottles were estimated from photographs of the bottles placed on study scales with their weights in grams visible. Full details on the procedures followed to determine consumption from partially empty bottles are provided in the protocol (https://osf.io/9u684/).

#### Secondary outcome

The mean time in days taken to consume each 1.5 l of wine during each intervention period with each bottle–glass combination, estimated from the start and finish dates reported on submitted photographs.

##### Covariates

1. Variables for (i) the bottle size used, (ii) glass size used, (iii) the bottle–glass interaction, (iv) the order in which households were allocated to the two bottle sizes (i.e. sequence effect) and (v) the intervention period in which a measurement was taken.
2. In‐home consumption of non‐study wine (in ml) by the household during each of the two 14‐day intervention periods, assessed by self‐reports on bottle labels.
3. Guest consumption of study wine (in ml) during each of the two 14‐day intervention periods, assessed by self‐reports on bottle labels.
4. Out‐of‐home consumption (in ml) by household members during each intervention period, assessed by self‐report.
5. Number of wine drinkers in household.
6. Duration (in days) of ‘usual behaviour’ period.
7. Baseline consumption (in ml) of wine per week, self‐reported.
8. Price per litre of all ordered wine.
9. Awareness of study aim, assessed in the end‐of‐study self‐report questionnaire.
10. Mitigating factors that could affect wine consumption, i.e. any noteworthy events or circumstances external to the study (e.g. illness, being away from home more than usual, etc.), self‐reported as having increased or decreased wine consumption during each intervention period.

#### Other measures

Demographic characteristics of households (mean household age, number of adults, annual household income) and of household representatives (age, gender, education, ethnicity), all self‐reported by participating household representatives.

### Statistical analysis

Demographic characteristics of households and household representatives completing the study were described [means (standard deviations: SDs); proportions (%)] and compared to those who enrolled into the study but did not complete it. Unadjusted summaries of consumption were also calculated for: (i) each bottle–glass condition; and (ii) each bottle–glass condition × period.

#### Primary analysis

The study was explanatory, rather than pragmatic, in nature. Pragmatic trials are often analysed according to the intention‐to‐treat principle [26]. In addition, it was considered highly unlikely that any dropout in the currently study was due to the assigned conditions. The primary analysis therefore was per‐protocol—i.e. households completing both interventions periods, excluding those violating the protocol.

A mixed‐effects regression analysis (adjusted effects) was used to predict total household wine consumption (in ml) at 14 days from the study start date with each bottle condition (i.e. 75 or 37.5‐cl) and glass condition (i.e. 290 versus 350 ml), fitting household as a random factor and potentially controlling for previously stated and pre‐specified covariates. Standard cross‐over design covariates also included in the model were variables for the order in which the two bottle sizes were purchased and period (i.e. first or second intervention periods). All regression model diagnostics were checked [i.e. residual, quantile–quantile (QQ) and influence plots] and were satisfactory. Model comparisons were was based on Akaike information criterion (AIC) values [27].

Four sets of sensitivity analyses were conducted by separately adding the following to the analysis: (i) all households that were randomized, i.e. intention‐to‐treat analysis, (ii) all households that completed the study, including those that violated the protocol, (iii) a variable for whether or not the study aims were guessed, and (iv) a per‐week variable (−1, 0, 1) for self‐reported mitigating factors influencing consumption which was aggregated over each period.

In line with recommendations [28, 29], Bayes factors (BF) were estimated using a recommended distribution‐based calculator [30] using one‐tailed tests. To calculate BFs relating to glass size and the interaction between bottle and glass size, a priori Cohen’s *d*s were used (*d* = 0.39 for glass size and *d* = 0.22 for the interaction). Calculated BFs were used alongside *P*‐values for the primary outcome analyses, in conjunction with other relevant information, to aid interpretation of effects and enable assessment of the strength of the evidence for each hypothesis, while noting that there is no deterministic relationship between Bayes factors and *P*‐values. Interpretation and reporting of the results were in line with guidance recommending a shift away from binary interpretations of significance based on *P*‐values [31].

### Secondary analysis

For the secondary outcome, a cumulative sum for the total volume of wine consumed was calculated per day. This was used to calculate the number of days taken to drink: (i) 1.5 l, (and for households that drank more); (ii) from 1.5 to 3 l; (iii) from 3 to 4.5 l; and (iv) from 4.5 to 6 l. These were averaged to calculate the mean number of days to drink 1.5 l.

A mixed‐effects regression analysis was performed, to predict the time taken (in days) to consume each unit of 1.5 l of wine from each bottle size with each glass size, fitting household as a random factor, with the same covariates as for the primary analysis.

Data are available from the Open Science Framework here: https://osf.io/43pue.

## RESULTS

**FIGURE 3:**
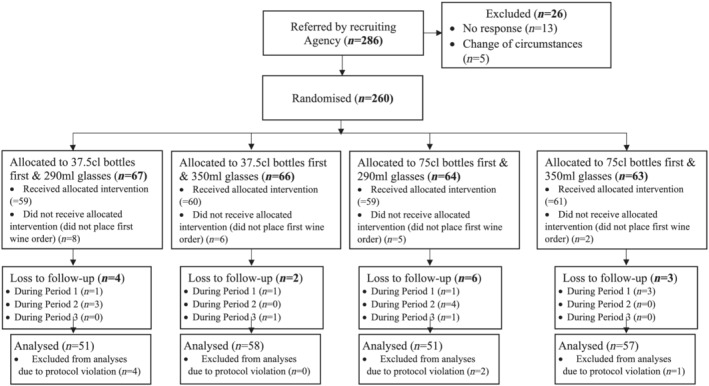
Flow of households through study

The flow of households through the study is shown in Figure 3. Two hundred and eighty‐six households were identified by the research agency, 278 of which were eligible to participate and 260 were randomized. Of the 260 households randomized, 224 (86%) completed the study in full but seven violated the protocol (3%), resulting in 217 that were included in the primary analysis (83%). The characteristics of households completing the study per protocol and of their representatives—i.e. the individuals who consented to take part in the study and provided data on behalf of their households—are shown in Table 1. The households and their representatives were broadly comparable to consumers of alcohol in Britain, the majority of whom are white and of higher socio‐economic position [32]. The characteristics of households dropping out of the study are shown in the Supporting information (Appendix S3, Table S1).

**TABLE 1:** Characteristics of (1) households (adults) and (1) household representatives completing the study per protocol (*n* = 217).

(1) Households	
No. of adults (mean, SD)	2.2 (0.8)
Age (mean, SD)	41.4 (12.5) range = 20–87
No of wine drinkers (mean, SD)	2.0 (0.6)
No of 75‐cl bottles of wine consumed per week (mean, SD)	2.6 (1.2)
Sex (%, *n*)	
Female	51.8% (134)
Male	48.8% (126)
Annual household income (%, *n*)	
Under £15 k	2.3% (5)
£15–25 k	6.0% (13)
£25–35 k	6.9% (15)
£35–50 k	18.0% (39)
£50–70 k	25.3% (55)
Above £70 k	36.4% (79)
Prefer not to say	5.1% (11)

(2) Household representatives	
Age (mean, SD)	42.4 (13.6) range = 20–71
Sex (%, *n*)	
Female	49.8% (108)
Male	50.2% (109)
Highest level of education (n (%))	
Below A levels^a^	16.6% (36)
A levels or vocational training	19.4% (42)
Bachelor’s degree and above	63.6% (138)
Prefer not to say	0.5% (1)
Ethnicity (*n*, %)	
White	90% (195)
Black	1.8% (4)
Asian	2.8% (6)
Mixed	5.1% (11)
Other	0.5% (1)

^a^ A‐levels are equivalent to a US high school diploma, a French Baccalauréat or a German Abitur. SD = standard deviation.

Descriptive information regarding the primary and secondary outcomes and covariates according to bottle size and glass size are shown in Table 2.

**TABLE 2:** Study outcomes and covariates [unadjusted: mean (standard deviation; SD)] during each 14‐day intervention period by bottle and glass sizes (*n* = 217).

	290‐ml glass (*n* = 102)			350‐ml glass (*n* = 115)			Across glass size (*n* = 217)	
	37.5‐cl bottle	75‐cl bottle	Across bottle size	37.5‐cl bottle	75‐cl bottle	Across bottle size	37.5‐cl bottle	75‐cl bottle
Primary outcome								
Volume of study wine consumed (ml)	3835.8 (1545.3)	4000.3 (1619.2)	3918.0 (1580.9)	4194.7 (2328.4)	4161.2 (2347.6)	4178.0 (2332.9)	4026.0 (2002.5)	4085.2 (2033.4)
Secondary outcome								
Mean number of days taken to consume 1.5 l of study wine	5.31 (2.37)	5.06 (2.28)	5.18 (2.32)	5.40 (2.57)	5.21 (2.31)	5.31 (2.44)	5.36 (2.47)	5.13 (2.29)
Covariates								
(i) Baseline consumption (ml)	–	–	1926.5 (665.5)	–	–	1972.8 (1037.2)	1951.0 (880.6)	
(i) Baseline consumption (ml)
(ii) Non‐study wine consumption (ml)	–	–	19.9 (106.4)	–	–	9.8 (84.7)	14.5 (95.4)	
(iii) Guest consumption (ml)	–	–	163.0 (416.1)	–	–	150.1 (360.7)	156.2 (386.9)	
(iv) Out‐of‐home consumption (ml)	–	–	235.0 (884.2)	–	–	291.1 (1110.1)	264.7 (1008.3)	
(v) Number of wine drinkers (%)								
One	–	–	16 (16)	–	–	13 (11)	29 (13)	
Two	–	–	70 (69)	–	–	91 (79)	161 (74)	
Three	–	–	14 (14)	–	–	9 (8)	23 (11)	
Four	–	–	2 (2)	–	–	2 (2)	4 (2)	
(vi) ‘Usual behaviour’ period duration (days)	–	–	7.5 (6.9)	–	–	8.8 (8.3)	8.2 (7.7)	
(vii) Price (£) per litre	–	–	14.4 (3.6)	–	–	13.8 (3.4)	14.1 (3.5)	
(viii) Guess study aim: yes (%)	–	–	70 (74)	–	–	79 (75)	149 (74)	
(ix) Mitigating factors	–	–	−0.1 (0.6)	–	–	−0.1 (0.9)	−0.1 (0.8)	

### Primary outcome: volume of wine consumed

The unadjusted difference in volume of wine consumed per household per 14‐day period when drinking from 37.5‐cl (smaller) bottles compared to 75‐cl (larger) bottles was −59.2 ml (smaller bottles: 4026.0 versus larger bottles: 4085.2 ml; Table 2). When drinking from 290‐ml (smaller) glasses compared to 350‐ml (larger) glasses this was −260.0 ml (smaller glasses: 3918.0 ml versus larger glasses: 4178.0 ml). When using smaller bottles with smaller glasses the difference in consumption was −325.4 ml compared to when using larger bottles with larger glasses (smaller bottles and glasses: 3835.8 ml versus larger bottles and glasses: 4161.2 ml) (Table 2).

After accounting for pre‐specified covariates, the difference in consumption per 14‐day period of drinking from smaller bottles compared to larger bottles was −145.7 ml (95% confidence intervals (CI = −335.5 to 43.7 ml) (Table 3). This equates to a −3.6% difference (95% CI = −8.3 to 1.1%). In interpreting the main effect of bottle size we considered the confidence intervals, which include both a possible substantial effect on reducing consumption and a marginal effect on increasing consumption, the associated *P*‐value (*P* = 0.137, Table 3) and the BF of 2.00. The BF indicated anecdotal evidence [28] for smaller bottles having an effect, suggesting that these data are approximately twice as likely to occur under the model including an effect for bottle size rather than the model without it.

**TABLE 3:** Mixed‐effect regression model estimates (95% CI) for volume (ml) of wine consumed per 14‐day period (*n* = 217)

	Estimate (SE)	*t*‐Value	*P*‐value	95% CI for estimate	
				Lower	Upper
Intercept	1129.89 (313.16)	3.61	< 0.001	526.38	1732.93
Bottle size 75 cl (ref: 37.5 cl)	145.66 (97.58)	1.49	0.137	−43.66	335.52
Glass size 350 ml (ref: 290 ml)	253.26 (136.85)	1.85	0.065	−10.17	516.94
Intervention order (ref: 75 cl first)	−302.97 (120.01)*	−2.53	0.012	−533.91	−72.04
Intervention period (ref: period 1)	−358.22 (66.32)**	−5.40	< 0.001	−487.04	−229.23
Baseline consumption (ml)	1.93 (0.07)**	28.20	< 0.001	1.8	2.06
Non‐study wine consumption (ml)	0.04 (0.23)	0.16	0.872	−0.4	0.47
Guest consumption (ml)	0.66 (0.13)**	5.24	< 0.001	0.42	0.91
Out‐of‐home consumption (ml)	−0.14 (0.04)**	−3.23	0.001	−0.23	−0.06
Number of wine drinkers (ref: 2)					
One	39.69 (179.25)	0.22	0.825	−305.22	384.61
Three	−108.72 (201.28)	−0.54	0.590	−496.02	278.78
Four	−675.68 (443.26)	−1.52	0.129	−1528.63	177.26
Log (‘Usual behaviour’ period duration (days) + 1)^†^	−378.93 (67.11)**	−5.65	< 0.001**	−508.06	−249.8
Price (£) per litre	1.14 (14.32)	0.08	0.937	−26.46	28.82
Bottle–glass interaction (ref: 37.5 cl & 290 ml)	−149.79 (131.54)	−1.14	0.256	−406.03	105.39

* Significant at the *P* < 0.05 level;

** Significant at the *P* < 0.01 level.

^†^ Skewed data were transformed.

Abbreviations: CI, confidence interval; SE, standard error.

The adjusted difference in consumption per 14‐day period of drinking from smaller glasses compared to larger glasses was −253.3 ml (95% CI = −516.9 to 10.2 ml). This equates to a −6.5% difference (95% CI = −13.2 to 0.3%). The CIs include both a possible substantial effect on reducing consumption and a marginal effect on increasing consumption. The associated *P*‐value for a main effect of glass size was *P* = 0.065 (Table 3). The BF was 2.96, indicating anecdotal, but borderline substantial, evidence for smaller glasses having an effect, suggesting that these data are almost three times more likely to occur under the model including an effect for glass size, rather than the model without it.

**FIGURE 4:**
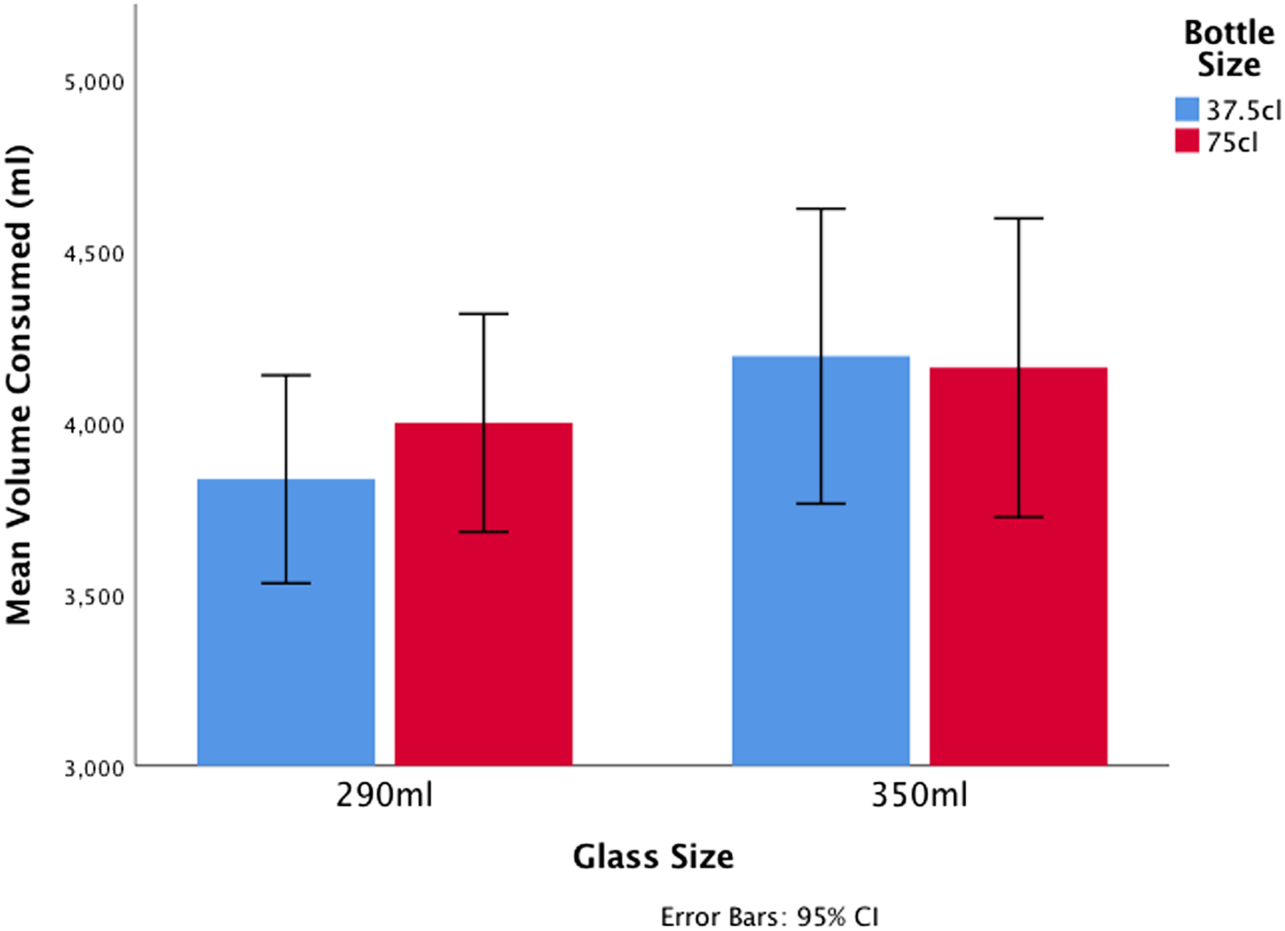
Wine consumed [(ml) mean] by glass and bottle sizes (observed values) [Color figure can be viewed at wileyonlinelibrary.com]

The adjusted difference in consumption per 14‐day period of using smaller bottles with smaller glasses compared to larger bottles with larger glasses was −249.1 ml (−6.5%) (95% CI = −519.6 to 21.2 ml; −13.6 to 0.6%) (Figure 4). The CIs include both a possible substantial effect on reducing consumption and a marginal effect on increasing consumption. The associated *P*‐value for a main effect of glass size was *P* = 0.077. The *P*‐value associated with the interaction effect was *P* = 0.26 (Table 3). The BF was 0.28, indicating substantial evidence for no interaction effect.

Of the set of pre‐specified covariates included in the statistical model, there was evidence of an effect of intervention period, with households drinking 358.2 ml (95% CI = 229.2–487.0, *P* < 0.001) less wine during the second study period compared to the first (Table 3; Supporting information Appendix S4: Figure S1). There was also a significant main effect of bottle size order, with households purchasing larger bottles first, consuming 303.0 ml (95% CI = 72.0–533.9, *P* = 0.012) less wine per 14‐day period compared to households purchasing smaller bottles first (Table 3; Supporting information Appendix S4: Figure S2 and Table S2). There were also significant main effects of baseline consumption, guest consumption, out‐of‐home consumption and the duration of the ‘usual behaviour’ period. More wine was consumed per 14‐day study period when households’ self‐reported baseline wine consumption was higher (a 1.93‐ml increase per 14‐day period for each 1‐ml increase in baseline weekly consumption; 95% CI = 1.80–2.06, *P* < 0.001) or reported having guests who drank from their study wine (a 0.66‐ml increase for each 1 ml consumed by guests; 95% CI = 0.42–0.91, *P* < 0.001). Less wine was consumed per 14‐day study period when households reported drinking wine out of the home (0.14‐ml decrease for each 1‐ml consumed out of the home; 95% CI = −0.23 to −0.06, *P* = 0.001). Less wine was also consumed per study 14‐day period for each extra log‐day spent in the ‘usual behaviour’ period (−378.3 ml; 95% CI = −249.8.1 to −508.1, *P* < 0.001) (Table 3).

### Secondary outcome: consumption rate

After accounting for pre‐specified covariates, there was no evidence of an effect of bottle size (*P* = 0.530) or glass size (*P* = 0.548), or of an interaction between the two on the rate of consumption (*P* = 0.731) (see Supporting information, Appendix S5 and Table S3). Of the set of pre‐specified covariates included in the statistical model, there was evidence of effects of bottle size order, baseline consumption, guest consumption and the duration of the ‘usual behaviour’ period (see Supporting information, Appendix S5, Table S3).

### Sensitivity analyses for primary analyses

Results and conclusions were unchanged with three of the four pre‐specified sensitivity analyses: (i) intention‐to‐treat analysis, (ii) including households which violated the protocol and (iii) including a variable for whether or not the study aims were correctly guessed (see Supporting information, Appendix S6 for details).

Results differed from the primary analysis in a sensitivity analysis, which included a variable for self‐reported mitigating factors influencing consumption. In this analysis, the effect of glass size was larger, with 262.6 ml (6.7%) less wine consumed per 14‐day period when using smaller glasses compared to larger glasses (95% CI = 17.7–507 ml; 0.45–12.9%). Notably, the confidence intervals around this effect became narrower, more clearly suggesting a substantial effect on reducing consumption when this underlying effect was accounted for. The associated *P*‐value was *P* = 0.040 (see Supporting information, Appendix S6, Table S7). The effect of smaller bottles and glasses was also larger in this analysis, with 291.5 ml (20.0%) (95% CI = 40.1–543.2 ml; 1.1–14.2%) less wine consumed per 14 day period with this bottle–glass combination compared to using larger bottles and glasses, with CIs suggesting a substantive effect on reducing consumption. The associated *P*‐value was 0.026.

## DISCUSSION

Households consumed on average approximately 3.6% less wine when drinking from smaller than from larger bottles. They also consumed on average approximately 6.5% less wine when drinking from smaller than from larger glasses. When using smaller bottles and glasses together, households consumed on average approximately 6.5% less wine. The uncertainty surrounding these effects was greatest for bottle size. We used CIs, *P*‐values and Bayes factors to interpret the evidence for each of these effects, in line with guidance recommending a shift away from binary interpretations of significance based on *P*‐values [31] and the use of additional sources of statistical information [28, 29].

The observed effect of bottle size was in the hypothesized direction, and is consistent with the findings of a previous study in which households consumed 4.5% less wine at home from 50‐cl bottles compared to 75‐cl bottles [17]. Confidence intervals around estimated consumption from the two studies almost entirely overlap (−7.5 to −1.0% in the previous study, versus −8.3 to 1.1% in the current study), but in the current study these slightly overlapped no effect. In conjunction with other information available regarding this effect, we cannot therefore be certain whether results are consistent with the hypothesis that smaller bottles reduce wine consumption at home. This may reflect a valid finding of there being no effect or that the current study was underpowered to detect a true effect of bottle size. The study was powered to detect an effect based on the observed effect of 50‐cl bottles [17], which may be larger than any effect of drinking from 37.5‐cl bottles. Smaller bottles—whether 50 or 37.5 cl—might reduce consumption by making additional wine intake more effortful [16], or reflecting the tendency for people to consume a specific number of units—such as bottles—in any one episode of consumption regardless of container size [33]. Smaller bottles might also increase consumption by reducing barriers to consumption that are present for larger sizes [19], or as a result of being considered too small. The latter two possibilities might be more likely with the use of 37.5‐cl than with 50‐cl bottles. The effect, therefore, on consumption of 37.5‐cl bottles might be smaller or less consistent than that of 50‐cl bottles. In addition, the observed effects of intervention period and bottle size order might have reduced the power to detect the predicted effect, while the order effects may have inflated the overall bottle comparison estimate. The effects of intervention period and bottle size order were assumed to be null when estimating the required sample size for the study. In sum, considerable uncertainty remains about the impact of consuming wine at home from 37.5‐cl compared with 75‐cl bottles, which precludes drawing clearer or more definitive conclusions. Field studies with greater power are needed to more reliably estimate the impact of 37.5‐cl bottles on wine consumption in homes.

The impact of glass size alone and in combination with bottle size were also in the hypothesized direction. Although the confidence intervals from the primary analysis around the effects of glass size and glass size in combination with bottle size included both possible meaningful effects on reducing consumption, they also included very small effects on increasing consumption. The Bayes factors associated with the primary analysis indicated that the evidence for an effect of glass size was borderline substantial [28]. The results of three sets of sensitivity analyses supported the presence of a glass size effect, and a combined effect of using smaller glasses and bottles, with confidence intervals suggesting a substantive effect on decreasing consumption or only a small effect on increasing consumption. The analyses suggesting the latter included non‐completers and/or protocol violators. The strongest evidence for a decrease in consumption, both for the glass size effect and the effect of smaller glasses and bottles, derived from repeating the primary analysis (i.e. households completing the study per protocol) to include a variable for whether participants reported any mitigating factors perceived to have affected their wine consumption. This analysis arguably provides the most valid estimate of the impact of bottle and glass size, as it controlled for factors most likely to have affected the primary outcome, such as illness or being away from the household and thus preventing consumption. The results of the fourth sensitivity analysis, which took into consideration whether households had guessed the study aims, did not support an effect of glass size.

The probable effect of smaller glasses on wine consumption in homes is in keeping with existing evidence of the impact of glass size in licensed premises and laboratory settings. A recent mega‐analysis found that wine served in larger glasses increased the volume of wine sold, and therefore consumed, in restaurants but not in bars [20]. Consistent with this, a recent laboratory study found that less wine was poured into smaller glasses compared to larger glasses [21], suggesting that smaller glasses might reduce consumption by reducing amounts that are self‐poured.

The current study did not find evidence for an interaction between bottle size and glass. This is consistent with the findings of the aforementioned laboratory study, which found no significant interaction effect when participants were asked to pour wine into differently sized glasses from differently sized bottles [21]. The effects might, however, be different when multiple servings are permitted and poured amounts are consumed, as was the case with the current study. Reflecting this, there was some evidence for an effect in reducing consumption when using smaller bottles and glasses compared with larger bottles and glasses.

The main strength of the current study is that it provides the first estimate, to our knowledge, of the impact on wine consumption in homes from 37.5‐cl bottles using glasses of different capacities, singly and in combination with different‐sized wine bottles. Further strengths include the study design and procedures, which minimized a number of possible biases through, for example, using a robust method to assess consumption and rigorous consent procedures to achieve a high retention rate.

The study had several limitations. First, the study power to detect bottle or glass size effects was lower than intended due to unanticipated intervention period and bottle size order effects. Period effects might have been the result of fatigue with drinking the study wine or seasonality and are expected to have lowered the power of the study to detect a bottle size effect. The observed order effects may have inflated the overall bottle comparison estimate. This was despite an attempt to mitigate these by allowing a usual behaviour ‘washout period’ of up to 3 weeks between intervention periods. Secondly, consumption of alcoholic beverages other than wine was not assessed in the study. It is not known, therefore, whether households compensated for any reduced wine consumption by drinking more of other alcoholic beverages. Thirdly, the generalizability of the results post‐Covid‐19 is unknown. Most data were collected when licensed premises in England were closed. As a result, wine consumption during the study might not reflect consumption during non‐pandemic times. The households and their representatives taking part in the present study were broadly comparable to consumers of alcohol in Britain [32], being predominantly white, of higher education and income. The generalizability of the results, therefore, to minority ethnic groups, of higher deprivation and of older age is unknown. Further research is needed to address these limitations as well as a number of uncertainties. Future studies should rely upon larger, more diverse samples to elucidate any impact of 37.5‐cl bottles and smaller glasses on wine consumption and assess the generalizability of findings to other bottle and glass sizes, populations and contexts. Importantly, future studies should aim to assess the sustainability of any effects that smaller wine bottles and glasses have on consumption beyond the time‐period assessed in the current study to assess whether effects are maintained over time.

The size of wine glasses has increased during the last three centuries, dramatically so during the last three decades [34]. If the effects of wine glass size on consumption are proved reliable with effects sustained over time, reducing the size of wine glasses used in homes could contribute to policies for reducing alcohol consumption. These could include pricing glasses according to capacity, which could increase the demand for smaller glasses. Regulating glass size—alongside serving size—in licensed premises is also a possibility, which could shift social norms for what constitutes an acceptable size of glass for use outside as well as at home [34]. Were an effect of smaller bottles—in particular, those of 37.5‐cl capacity—to be more certain, possible policies for shifting purchasing and consumption to smaller bottle sizes might include increasing their availability and affordability. The latter might involve proportionate pricing achieved through taxation. The results of this study are potentially applicable to other types of containers and glasses, with implications for policies to reduce consumption of other alcoholic drinks, including beer.

In conclusion, drinking wine at home using smaller glasses may reduce consumption. Greater uncertainty remains around the possible effect of drinking from smaller bottles with or without smaller glasses. Further studies are warranted to improve the precision of the estimated effect sizes of these interventions given their potential to contribute to comprehensive alcohol control policies.

## DECLARATION OF INTERESTS

None.

## AUTHOR CONTRIBUTIONS

TMM, EM, and GJH conceptualized and designed the study. EM coordinated the study and led on data collection and cleaning with MV and JF. MP led on the statistical analysis. EM, MP, GJH, and TMM drafted the manuscript with all authors providing critical revisions. TMM is a guarantor.

## Supporting information


**Appendix S1** ‐Instructions sent to Participants
**Appendix S2** – Study wine list
**Appendix S3** – Characteristics of households dropping out
**Appendix S4** – Primary analysis ‐Additional plots and tables
**Appendix S5** ‐ Secondary analysis ‐ Impact of covariates
**Appendix S6** ‐ Sensitivity analyses
