# Supplementary material for: Impact of wine bottle and glass sizes on wine consumption at home: a within‐ and between‐ households randomized controlled trial
Source: Addiction. 2022 Aug 2;117(12):3037–48. doi: 10.1111/add.16005 (PMC9804259; doi:10.1111/add.16005)
Supplement: Supplementary file 1 — Appendix S1 ‐Instructions sent to Participants Appendix S2 – Study wine list Appendix S3 – Characteristics of households dropping out Appendix S4 – Primary analysis ‐Additional plots and tables Appendix S5 ‐ Secondary analysis ‐ Impact of covariates Appendix S6 ‐ Sensitivity analyses [file ADD-117-3037-s001.docx]

**Supplementary Material**

**Index**

**S1 -Instructions sent to Participants** page 2

**S2 – Study wine list** page 13

**S3 – Characteristics of households dropping out** page 24

**S3 – Primary analysis – ~~Impact of covariates~~ Additional plots and tables** page 25

**S4 – Secondary analysis – Impact of covariates** page 26

**S5 – Sensitivity analyses** page 28

**S1 -Instructions sent to Participants**

Participant ID:

Dear

**Title of Study:** ‘Impact of bottle size and glass size on people’s experiences of consuming wine’

Thank you for completing the first step of the study *i.e.* placing your first wine order from Tanners and sending us the order confirmation.

You will receive £20 for completing this step and £8.50 to compensate for any wine delivery costs.

With this letter, you will find a study information sheet and some **labels to stick to your wine bottles** during Study Period 1 and Study Period 3.

You will also receive a set of **wine glasses** to use during the study and a set of **study scales** with batteries.

Below are instructions on what you will need to do during the study.

**You can also find all the information in this letter in two short study videos:**

Part I (study overview): <https://youtu.be/XzAaqBVK6d0>

Part II: (completing study tasks): <https://youtu.be/NSOynbrJaSA>

lin

**The study has three study periods, shown below:**

**
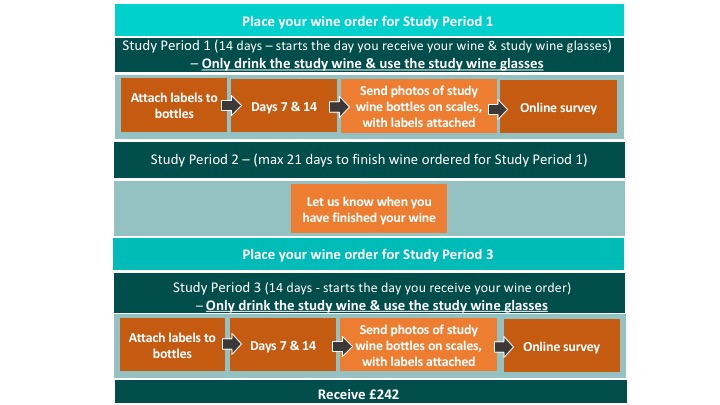
**

**When can I start the study?**

**You need to complete four steps**:

1. Send us a **photograph** of your **study wine bottles** as soon as they arrive to confirm that you have received them
2. **Confirm** that you have received your **study labels**
3. Send us a **photograph** of the **study wine glasses** as soon they arrive to confirm that you have received them
4. Send us a **photograph** of the **study scales** as soon they arrive to confirm that you have received them.

Confirmations and photographs should be sent either by **email**: [WBResearch@medschl.cam.ac.uk](mailto:WBResearch@medschl.cam.ac.uk) or by **WhatsApp**: **07377978968**

Once you have completed these four steps, **we will let you know when you can begin the study**


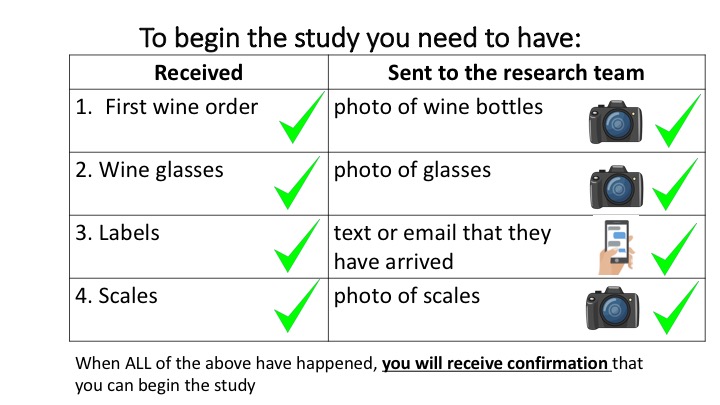


**Please do not start drinking your study wine before you have**

**received your study wine glasses and study scales and we have confirmed that you can begin the study**

**What to do when you receive your study wine glasses**

(refer to video Part II <https://youtu.be/NSOynbrJaSA>)

*If you haven’t already,* ***you should receive a set of wine glasses to use during the study****.*

When you receive the study wine glasses, **please follow these steps**:

1. Remove them from their packaging and **send a photograph to the research team - either by email or WhatsApp.**
2. Place them in the cupboard or on the shelf where you normally store your wine glasses.
3. Keep them accessible. If possible, place the study wine glasses in front of your existing wine glasses by pushing the existing wine glasses to the back of the cupboard or shelf.
4. If there is no available space where you normally store your wine glasses, please remove your existing wine glasses and store them elsewhere during the study, preferably out of sight.

**STUDY PERIOD 1**

**Study Period 1** starts when the study team **confirms that you can begin (see page 2 above).**

**During this time, you will need to:**

- Stick the study labels to your study wine bottles (one label per bottle).
  - These labels are enclosed in the envelope labelled “Study Period 1 Labels”.
  - Instructions on how to position the labels on the bottles are on Page 7
- Use the labels to record:

1. the date each bottle was opened and finished
2. the number of people, including any guests, who drank from each bottle

*Instructions on how to complete the labels are on Page 7*

- Drink the study wine as you would normally drink wine at home
- **Drink only the study wine**
- Drink wine **only from the study wine glasses**
- Send the study team photographs of each study wine bottle (unopened, opened but not finished, and empty) placed on the study scales on two occasions: 7 days and 14 days after the beginning of Study Period 1.

*Instructions on how to take these photographs are detailed on Page 8*

- Send the study team photographs of the wine glasses you were using on Day 7 and Day 14 after the beginning of Study Period 1.
- Complete a short online questionnaire on two occasions: 7 days and 14 days after the beginning of Study Period 1. The study team will email or WhatsApp the link to these questionnaires

**You need to send the study team your photographs on Day 14 to be able to continue with the next study periods and receive full compensation.**

**Please let us know during this period** if:

- you are running low on wine and need to place an additional order to last you for Study Period 1
- if any of your wine glasses have broken and need replacing.

**STUDY PERIOD 2**

**Study Period 2** is a gap between Study Period 1 and Study Period 3, lasting a maximum of 3 weeks.

This is to give you time to finish any remaining wine from your first wine order before placing your second wine order.

You should place and receive your second wine order within 21 days of finishing Study Period 1.

**During this time, you will need to:**

- Notify us by email/WhatsApp as soon as your wine is finished or close to finishing (*i.e.* you have opened your last bottle).
- Visit our study website to choose your wines, just as you did for Study Period 1. We will send you the link to our website. We will inform you of how many bottles you will need to order and in which size
- Send us confirmation of your second wine order

1. Send us a photograph of your wine bottles as soon as they arrive to confirm that you have received them

**Your second wine order should be placed and your wine received within 21 days (3 weeks) of finishing Study Period 1.**

**If you have not received your second wine order in this timeframe, your participation will be ended and you will receive no further compensation.**

If we have not heard from you within two weeks of the end of Study Period 1, we will contact you to ask you about any leftover wine you have.

**STUDY PERIOD 3**

**Study Period 3** starts when the study team **confirms that you can begin**

**You will now need to repeat the procedures you followed during Study Period 1:**

- Stick the study labels to your study wine bottles (one label per bottle).
- These labels are enclosed in the envelope labelled “Study Period 3 Labels”.
- Instructions on how to position the labels on the bottles are on Page 7
- Use the labels to record:

1. the date each bottle was opened and finished
2. the number of people, including any guests, who drank from each bottle

*Instructions on how to complete the labels are on Page 7*

- Drink the study wine as you would normally drink wine at home
- **Drink only the study wine**
- Drink wine **only from the study wine glasses**
- Send us a photograph of each study wine bottle (unopened, opened but not finished, and empty) placed on the study scales on two occasions: 7 days and 14 days after the beginning of Study Period 3.

*Instructions on how to take these photographs are detailed on Page 8*

- Send us a photograph of the wine glasses you were using on Day 7 and Day 14 after the beginning of Study Period 3.
- Complete an online questionnaire on two occasions: 7 days and 14 days after the beginning of Study Period 3. We will email or WhatsApp you the link to these questionnaires

**You need to send us your photographs on Day 14 to be able to receive full compensation.**

During Study Periods 1 and 3, we will send you reminder WhatsApp messages or emails to send us your photographs on the morning of the day they are due. If we don’t hear from you, we will send you follow-up reminders.

**Compensation**

You will receive a **maximum of £242**, **if you complete all aspects of the study**.

Below is a breakdown of how much you will receive for each task you complete.

You will receive payment from Roots Research via PayPal or bank transfer on two occasions: within two weeks of completing Study Period 1 and within two weeks of completing the study.

**STUDY PERIOD 1**

*Ordering wine and providing confirmation of order:* £20

*Wine delivery costs*: £8.50

*Sending photograph of wine glasses upon their receipt*: £10

*Sending photographs of all bottles of ordered wine on:*

- Day 7: £15
- Day 14: £40

*Sending photographs of wine glasses used:*

- Day 7: £5
- Day 14: £15

**STUDY PERIOD 2**

*Ordering wine and providing confirmation of order:* £40

*Wine delivery costs*: £8.50

**STUDY PERIOD 3**

*Sending photographs of all bottles of ordered wine on:*

- Day 7: £20
- Day 14: £40

*Sending photographs of wine glasses used:*

- Day 7: £5
- Day 14: £15

There will be no additional compensation for the wine purchases made during the study.

**How to complete the study labels** (refer to video Part II <https://youtu.be/NSOynbrJaSA>)

1. Use a ballpoint pen (blue or black) to fill in the labels to avoid your answers rubbing off or smudging.
2. Try to keep a note of any wine that you give to anyone not part of your household – *i.e.* guests - and any non-study wine you and / or your household members drink whilst at home.
3. Align the left side of the study label with the left side of the front manufacturer’s label.

**Attach a label on each bottle you receive and complete it as follows**:

1. Fill in the date you opened this bottle and the date you finished it.

**The label**

| Date bottle was opened: |  |
| --- | --- |
| Date bottle was finished: |  |
| No. of household members who drank from this bottle:  : | |
| No. of non-household members (guests) who drank from this bottle: | |
| Amount drunk from this bottle by non-household members (guests):  *Small glass (125ml):*  *Standard glass (175ml):*  *Large glass (250ml):*  *Whole bottle:* | |
| Amount of non-study wine drunk at home by your household during above dates:  *Small glass (125ml):*  *Standard glass (175ml):*  *Large glass (250ml):*  *Bottle (75cl):* | |

1. How many people from your household, including yourself, drank from this bottle?
2. How many people not part of your household (i.e. guests), drank from this bottle?
3. If you had guests that drank from this bottle, add the number of each serving size they drank. For example:

Small glass (125ml): 1

Standard glass (175ml): 2

Large glass (250ml): 0

If your guests drank the whole bottle, add a “YES” next to the ‘Whole bottle’ option.

1. If any of your household members, including yourself, drank any non-study wine while at home, between opening and finishing this bottle, add the number drunk next to each option. For example:

Small glass (125ml): 0

Standard glass (175ml): 2

Large glass (250ml): 0

Bottle (75cl) :1


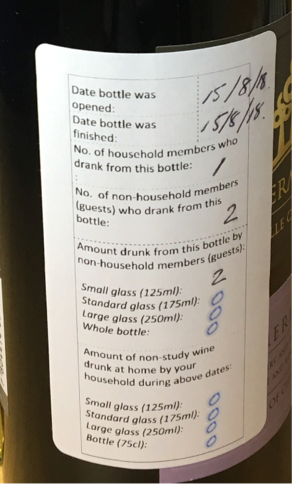


**Example of a completed label:**

**How to take study photographs (**refer to video Part II <https://youtu.be/NSOynbrJaSA>)

On Day 7 and Day 14 of Study Periods 1 and 3 you will need to send the study team photographs of:

- your study wine bottles (all bottles: unopened, opened but not finished, and empty);
- the wine glasses you were using on those days.

You need to send photographs according to these instructions:

Study Wine Bottles

1. **Place each bottle,** whether unopened, opened but not finished, or empty, **on the study scales and photograph each bottle so that both the bottle and the number on the scales (*i.e.* the weight of the bottle) is visible.**
   1. **Unopened bottles** should be photographed with their **tops on**
   2. **Opened** but not finished bottles **and empty bottles** should be **photographed with tops off**
2. **Photograph each study wine bottle separately.**

Do not take one photograph of all bottles together.

For example, if you order six bottles of wine, you should send us six individual photographs.

1. **Ensure that** the **entire bottle is clearly visible** – including its full base and **top**
2. Make sure the **study** **label** attached to each bottle **is clearly visible and readable,** or that we can zoom in and read it. If the label is not clearly readable in the photograph of the entire bottle, take an additional close-up photograph of just the label.
3. Make sure the **number on the scales (*i.e.* the weight of the bottle)** is **clearly visible and readable**.

If the weight of the bottle is not clearly readable in the photograph of the entire bottle, take an additional close-up photograph of just the weight of the bottle, making sure to tell us which bottle the weight corresponds to by reporting the number on the label.

1. Photographs must be **clear** and **NOT** **blurry**

Study Wine Glasses

1. **Take a photograph of the wine glasses you were using on Day 7 and Day 14** next to a bottle from your wine order
2. Take a photograph of **each type of wine glass** you used on Day 7 or Day 14 **separately**
3. The whole glass – *i.e.* from top to bottom – needs to be clearly visible in the photograph

Examples of acceptable and unacceptable photographs are shown on the next page.

**Examples of ACCEPTABLE photos**

**
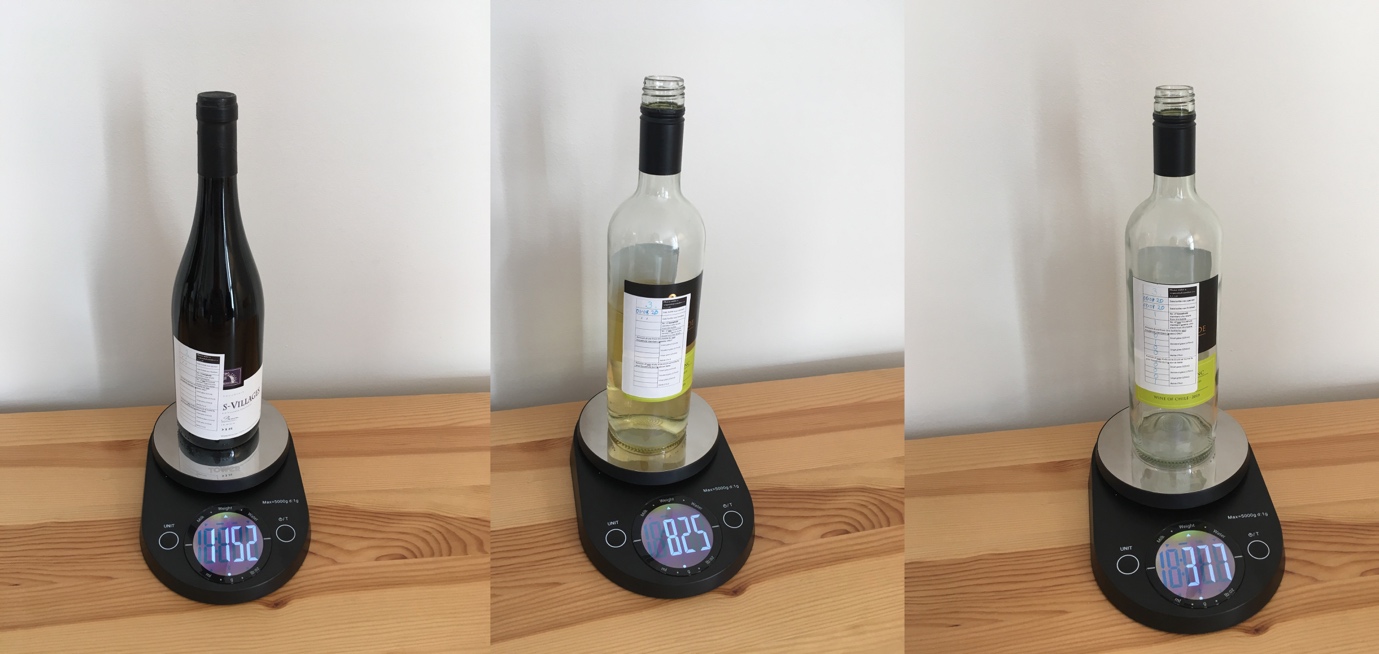

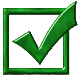

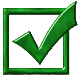

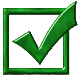
**

|  |
| --- |

- *Photographs are clear and not blurry.*
- *Both the study label and the weight of the bottle are clearly visible and readable when you zoom in.*
- *One bottle is presented per image.*
- *The entire bottle is visible.*
- *Opened but not finished bottles and empty bottles are photographed with their tops off.*

*~~
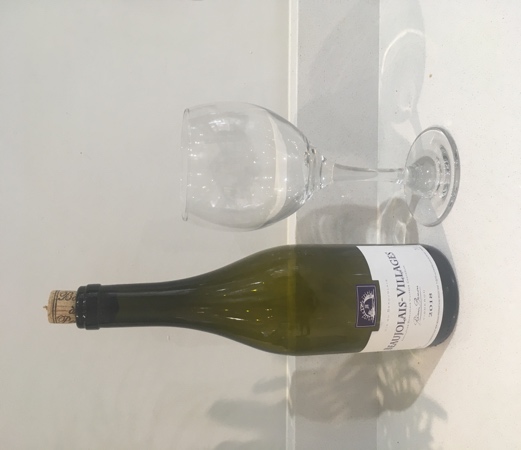
~~***
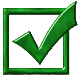
**

- *The wine glass is photographed next to a wine bottle.*
- *Only one wine glass is in the photograph.*
- *Both the entire wine bottle and the entire wine glass are clearly*

*visible.*

**
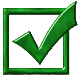
**

**Examples of UNACCEPTABLE photos:**

**
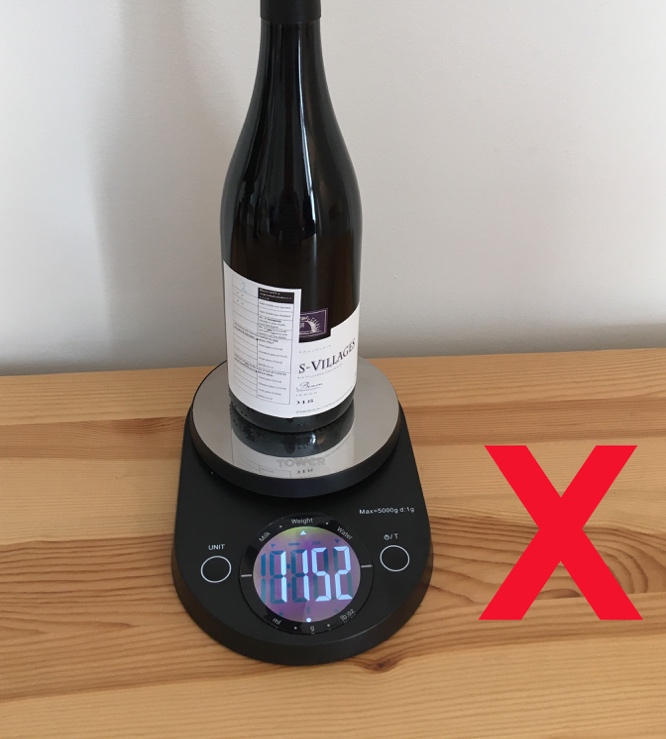

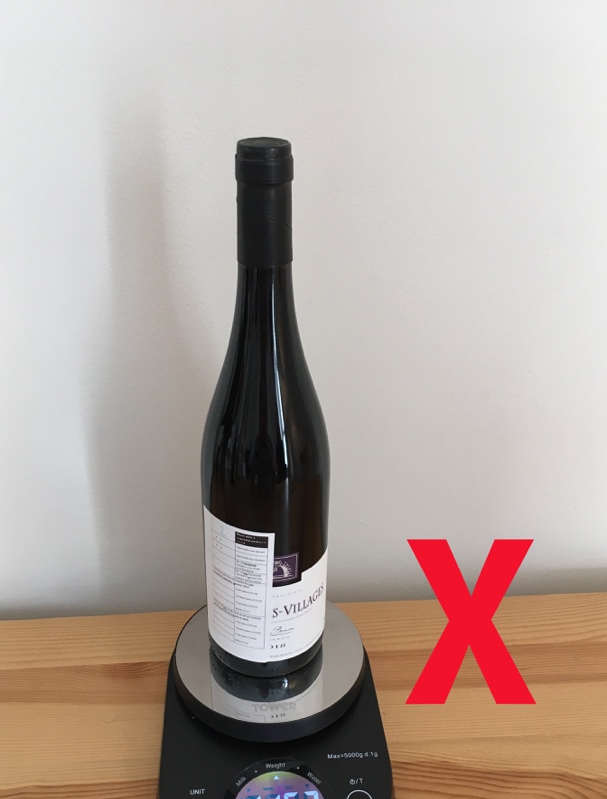
**

| **Problem:** *Number on scales not visible* **Problem:** *Entire bottle not visible*  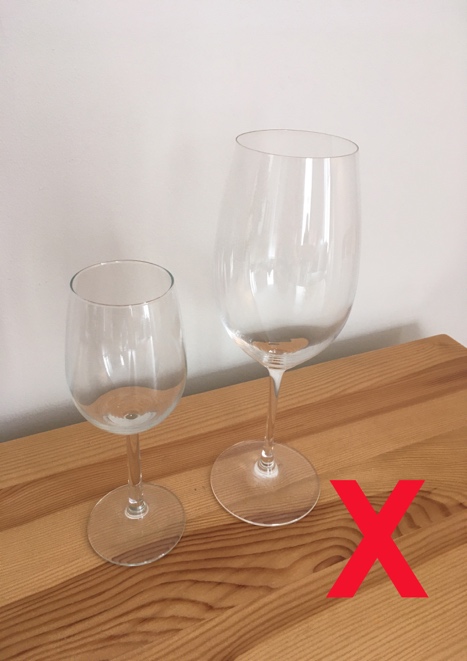  **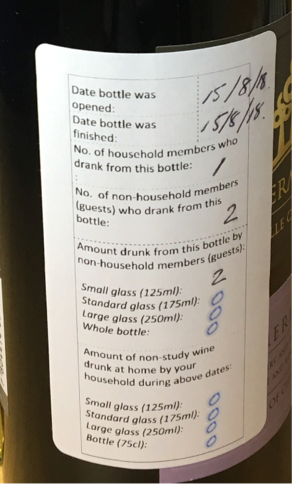**  **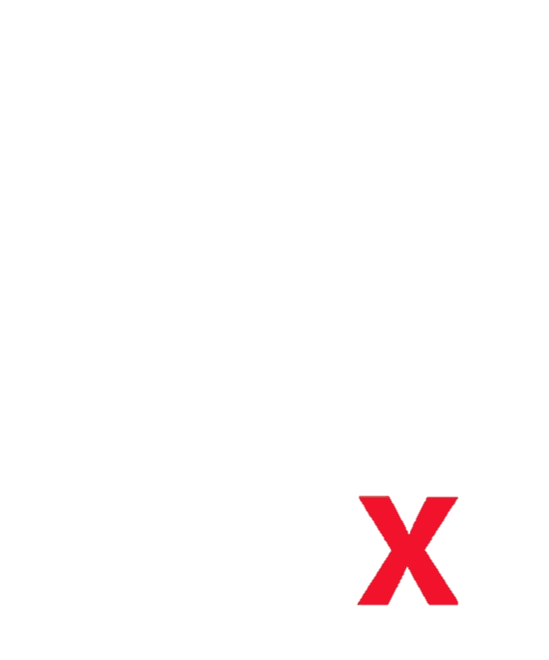**  **Problem:**   - *Each wine glass type not* - *photographed separately* - *Glasses not next to a wine bottle*   **Problem:**   - *Close-up of label only.* - *The entire bottle and number on the scales are not visible*   **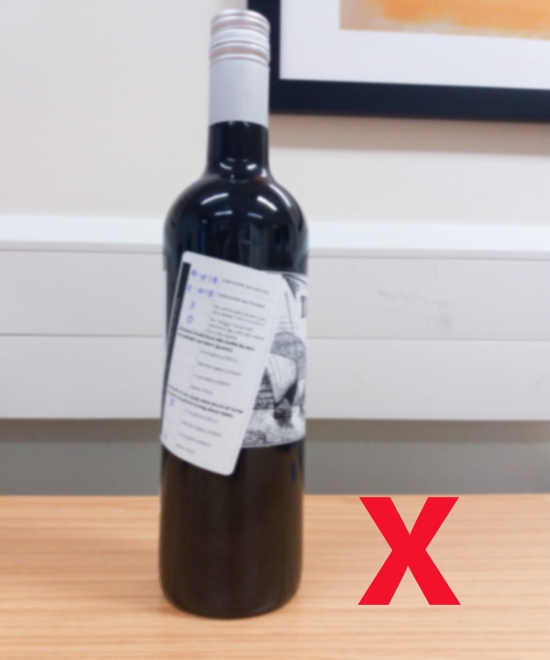**  **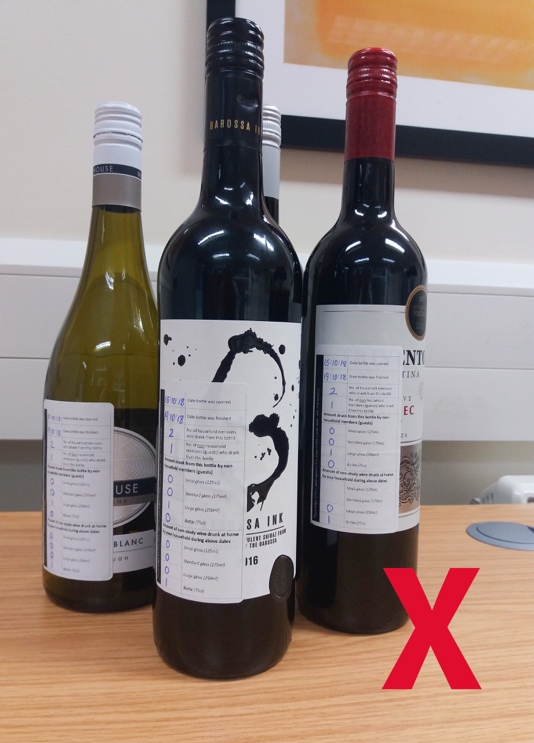**  **Problems:**   - *Image is blurry.* - *Bottle not placed on scale* - *Label not positioned correctly* |
| --- |
| **Problems**:   - *Many bottles in image – the bottles behind are not visible.* - *Bottles not placed on scales* |
|  |

If you have any questions about these instructions, or are unclear about anything else in the study,

please contact Eleni Mantzari, Project Manager, on **07377978968** or via email: [WBResearch@mesdchl.cam.ac.uk](mailto:WBResearch@mesdchl.cam.ac.uk)

You can also refer to the instructions in the two study videos:

Part I (study overview): <https://youtu.be/XzAaqBVK6d0>

Part II: (completing study tasks): <https://youtu.be/NSOynbrJaSA>

**How to send photos to the study team via email or WhatsApp**

**Email pictures to** [**WBResearch@mesdchl.cam.ac.uk**](mailto:WBResearch@mesdchl.cam.ac.uk)**:**

- If you want to email the image to us, put your Participant ID in the Subject Line (found at the top of this letter), so that we can check that the images we receive are yours.

**WhatsApp pictures to 07377978968:**

- If you want to message the image to us, before hitting ‘send’ please add your Participant ID to the message (found at the top of this letter) so that we can check that the images we receive are yours.

**Further instructions for how to send a photo taken on your mobile phone**

1. Depending on the type of phone you have, after taking a photo you may return to your camera and your gallery will have the new image. If this happens, please select the picture you just took (it may be at the top or bottom of your screen) in order to go to the gallery.
2. When you’re in your gallery, you may need to click the image you want to send to us again, in order to see the ‘share’ symbol (which could look like this
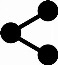

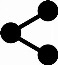

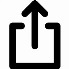
 [
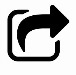
](https://www.bing.com/images/search?view=detailV2&ccid=FgshjIvg&id=A481169811534ED054081C9385E4453BBE752814&thid=OIP.FgshjIvgPwYzzEoj1DU24AHaHa&mediaurl=http://www.free-icons-download.net/images/share-share-icon-63786.png&exph=512&expw=512&q=share+icon&simid=608053945616043972&selectedIndex=24)) or an ‘options’ symbol (which could look like this
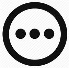
 ) at the top or bottom of your screen.
3. If you see the ‘share’ icon, click on it and then select the way you want to send the image to us – via email or WhatsApp.
4. If you see the ‘options’ icon, click on it and then select ‘Share via…’. You will then be able to select the way you want to send the image to us.
5. Your WhatsApp or email app will need to have access to your photos in order to directly send the image – so don’t worry if your phone asks for permission to do this.
6. When you have selected the way you want to send the image to us – the selected program will open.
   - If you want to email the image to us, enter [WBResearch@mesdchl.cam.ac.uk](mailto:WBResearch@mesdchl.cam.ac.uk) in the ‘To’ line and put your Participant ID in the Subject Line.
   - If you want to WhatsApp the image to us, enter our mobile number **07377978968** or add it to your Contacts List. Before hitting ‘send’ please add your Participant ID to the message.

If you have any questions about sending photos, please contact Eleni Mantzari on **07377978968** or via email [WBResearch@mesdchl.cam.ac.uk](mailto:WBResearch@mesdchl.cam.ac.uk).

**S2 – Study wine list^[[1]](#footnote-2)^**

This is a simplified version of what participants saw. Participants viewed separate wine lists for 75cl and 37.5l bottles and their versions included tasting notes, country of origin and links to the wine on the retailer website.

**RED WINE**

**
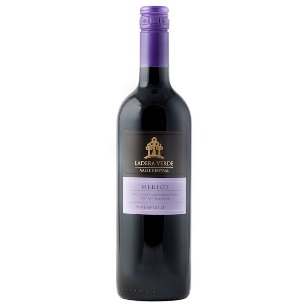
Ladera Verde Merlot, Valle Central 75cl: £7.50; 37.5cl: £4.25**

**Merlot - Product of Chile**

**ABV:** 13.5%


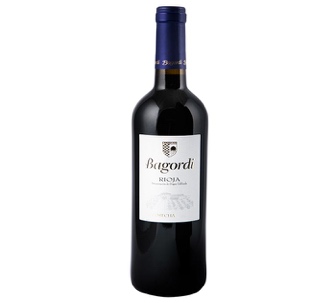
**Bagordi Tinto Cosecho, Rioja 75cl: £8.80; 37.5cl: £5.20**

**Rioja – Product of Spain**

**ABV:** 14%

**
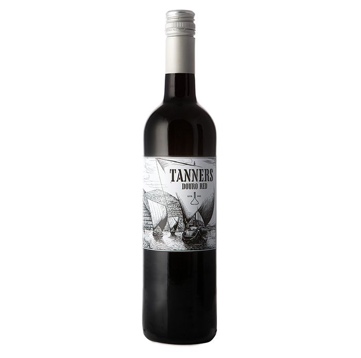
Tanners Douro Red 75cl: £8.80; 37.5cl: £5.40**

**Touriga Nacional – Product of Portugal**

**ABV:** 13.5%


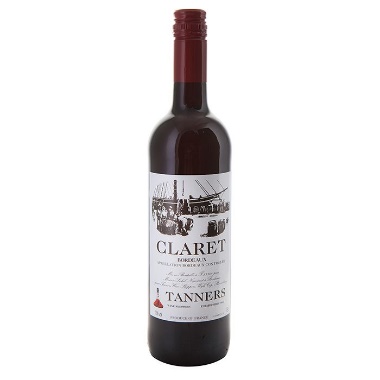
**Tanners Claret, Bordeaux 75cl: £9.50; 37.5cl: £5.70**

**Merlot/Cabarnet Sauvignon – Product of France**

**ABV:** 13.5%


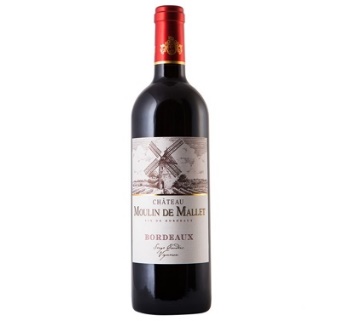
**Château Moulin de Mallet, Bordeaux 75cl: £9.70; 37.5cl: £6.30**

**Merlot – Product of France**

**ABV:** 14%


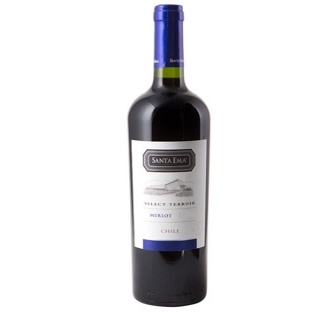
**Santa Ema 'Select Terroir' Merlot Reserva, Maipo 75cl: £9.80; 37.5cl: £5.70**

**Merlot – Product of Chile**

**ABV:** 13.5%


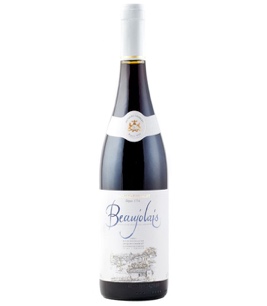
**Beaujolais, Jacques Charlet 75cl: £9.95; 37.5cl: £5.90**

**Gamay – Product of France**

**ABV:** 13%


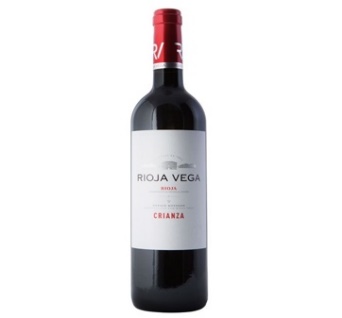
**Rioja Vega Crianza, Rioja 75cl: £10.90; 37.5cl: £6.40**

**Rioja – Product of Spain**

**ABV:** 14%


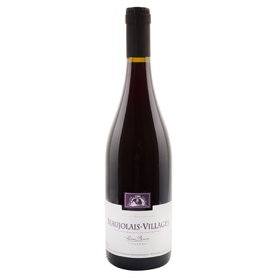
**Beaujolais-Villages, Rémi Benon 75cl: £12.50; 37.5cl: £7.70**

**Gamay – Product of France**

**ABV:** 12.5%


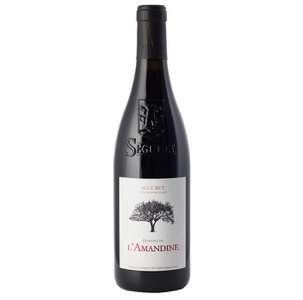
**Séguret, Côtes du Rhône-Villages, Domaine de L'Amandine 75cl: £12.70; 37.5cl: £7.60**

**Grenache – Product of France**

**ABV:** 14%


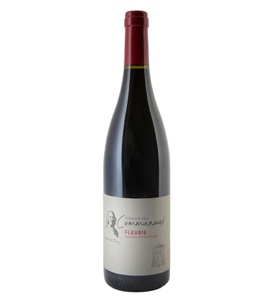
**Fleurie, Domaine des Communaux, Jaques Charlet 75cl: £13.80; 37.5cl: £7.95**

**Gamay – Product of France**

**ABV:** 13.5%


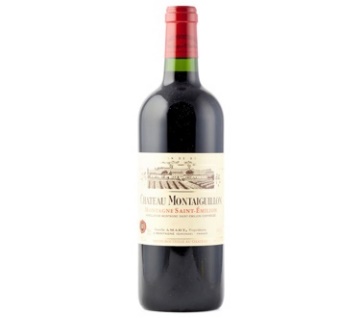
**Château Montaiguillon, Montagne Saint-Emilion 75cl: £16.90; 37.5cl: £8.90**

**Merlot – Product of France**

**ABV:** 13.5%


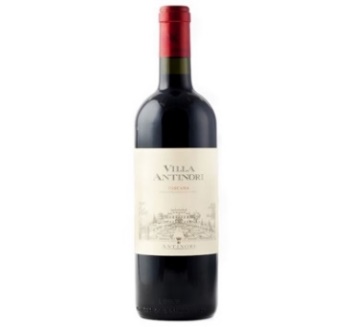
**Villa Antinori Rosso, Toscana 75cl: £22.50; 37.5cl: £13.90**

**Sangiovese/Brunello – Product of Italy**

**ABV:** 14.5%

**
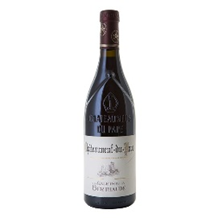
Châteauneuf-Du-Pape, Les Galets De La Berthaude, Roger Perrin 75cl; £23.50; 37.5cl: £13.80**

**Grenache – Product of France**

**ABV:** 15%


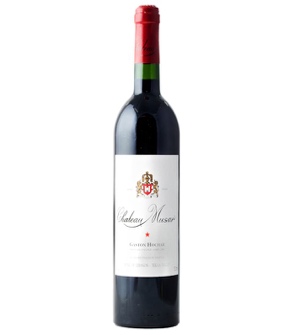
**Château Musar, Bekaa Valley, Gaston Hochar 75cl: £29.50; 37.5cl: £17.50**

**Cabernet Sauvignon – Product of Lebanon**

**ABV:** 14%


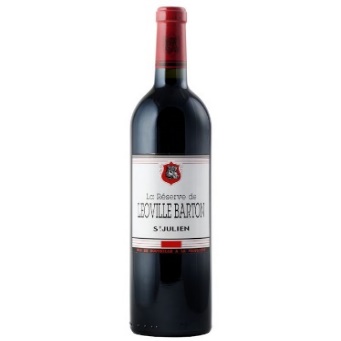
**La Réserve de Léoville Barton, Saint-Julien 75cl: £39.50; 37.5cl: £19.50**

**Cabernet Sauvignon – Product of France**

**ABV:** 13%

**WHITE WINE**


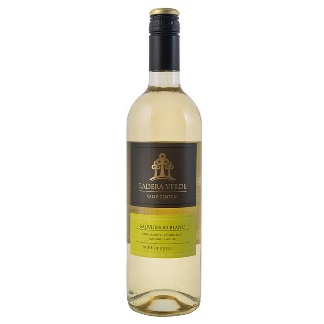
**Ladera Verde Sauvignon Blanc, Valle Central 75cl: £7.50; 37.5cl: £4.25**

**Sauvignon Blanc – Product of Chile**

**ABV:** 12%


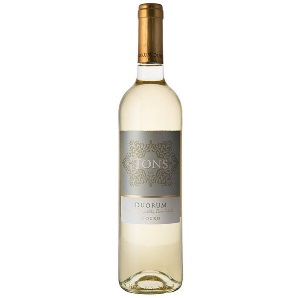
**Tons de Duorum, Douro Branco 75cl: £9.50; 37.5cl: £5.60**

**Viosinho – Product of Portugal**

**ABV:** 12.5%


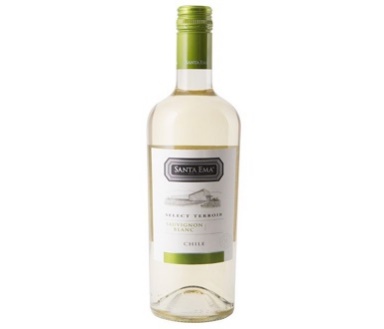
**Santa Ema 'Select Terroir' Sauvignon Blanc 75cl: £9.95; 37.5cl: £5.20**

**Sauvignon Blanc – Product of Chile**

**ABV:** 13%


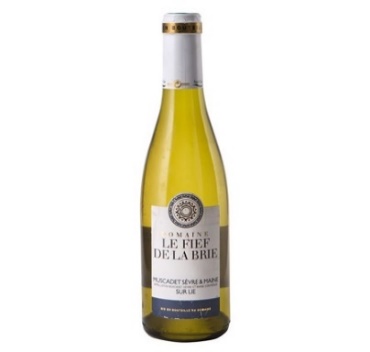
**Muscadet Sèvre-et-Maine Sur Lie, Domaine Le Fief de la Brie 75cl: £10.50; 37.5cl: £6.20**

**Muscadet – Product of France**

**ABV:** 12%


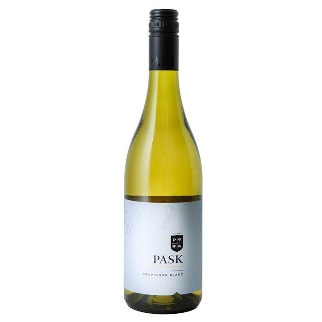
**Pask ‘Gimblett Gravels’ Sauvignon Blanc, Hawkes Bay 75cl: £10.95; 37.5cl: £6.20**

**Sauvignon Blanc – Product of New Zealand**

**ABV:** 12.5%


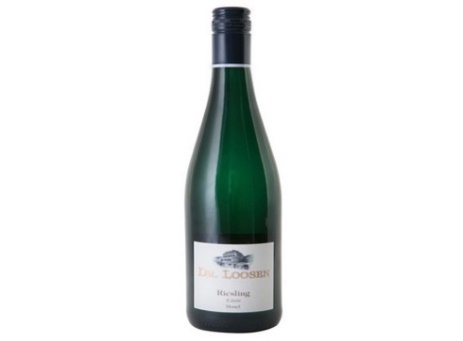
**Dr Loosen Estate Riesling, Mosel 75cl: £12.50; 37.5cl: £6.20**

**Riesling – Product of Germany**

**ABV:** 8.5%


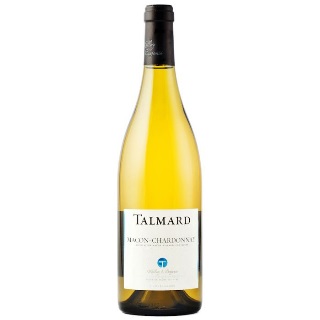
**Mâcon-Chardonnay, Mallory & Benjamin Talmard 75cl: £12.50; 37.5cl: £7.20**

**Chardonnay – Product of France**

**ABV:** 13.5%


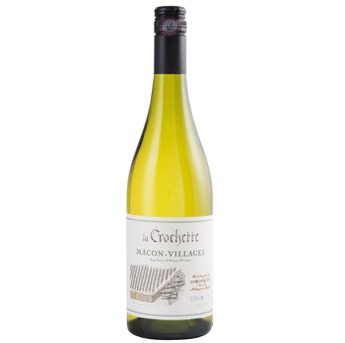
**Mâcon-Villages, La Crochette, Jean Loron 75cl: £13.50; 37.5cl: £7.90**

**Chardonnay – Product of France**

**ABV:** 13%


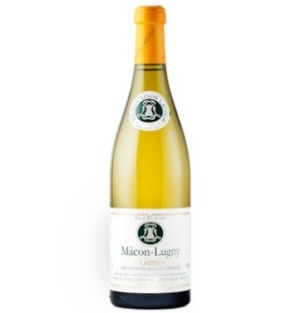
**Mâcon-Lugny, Les Genièvres, Maison Louis Latour 75cl: £14.50; 37.5cl: £7.95**

**Chardonnay – Product of France**

**ABV:** 13%


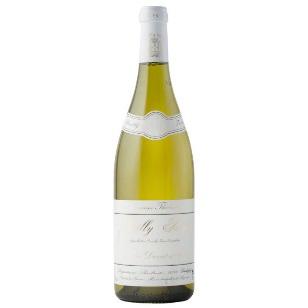
**Pouilly-Fumé, Domaine Thibault, André Dezat 75cl: £17.90; 37.5cl: £9.20**

**Sauvignon Blanc – Product of France**

**ABV:** 13%


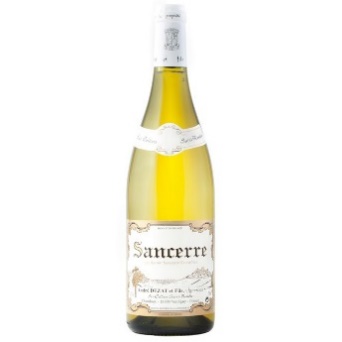
**Sancerre, André Dezat et Fils 75cl: £17.50; 37.5cl: £9.50**

**Sauvignon Blanc – Product of France**

**ABV:** 13%


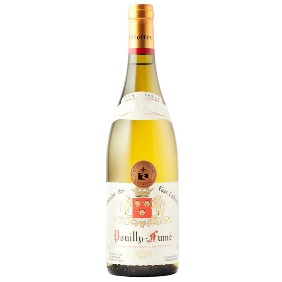
**Pouilly-Fumé, Domaine Des Fines Caillottes, Jean Pabiot 75cl: £17.50; 37.5cl: £9.50**

**Sauvignon Blanc – Product of France**

**ABV:** 13%

**Chablis 1er cru Côte de Léchet*,*Daniel*-*Etienne Defaix 75cl: £35; 37.5cl: £17.40**


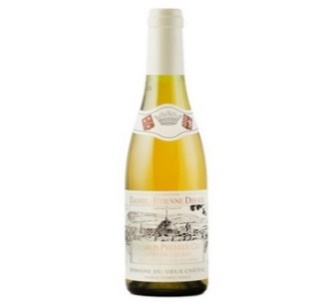


**Chardonnay – Product of France**

**ABV:** 13%

**S3 – Characteristics of households dropping out**

**Table S1:** Characteristics of (a) households (adults) and (b) household representatives dropping out of the study (n=32)

|  | **Overall (n=166)** |  |
| --- | --- | --- |
| 1. **Households** | |  |
| **No of adults (mean (sd))** | 1.9 (0.8) |  |
| **Age (adults in household) (mean (sd))** | 42.1 (12.2) |  |
| **Sex (mean % (sd))**  Female  Male | 56.8% (29.5)  43.2% (29.5) |  |
| **No of wine drinkers (mean (sd))** | 1.8 (0.8) |  |
| **No of 75cl bottles of wine consumed per week (mean (sd))** | 2.9 (1.9) |  |
| **Annual household income** **(n (%))** |  |  |
| £15-£25k | 5 (15.6%) |  |
| £25-£35 | 4 (12.5%) |  |
| £35-£50k | 8 (25%) |  |
| £50-£70k | 5 (15.6%) |  |
| Above £70k | 5 (15.6%) |  |
| Prefer not to say | 5 (15.6%) |  |
| 1. **Household representatives** | |  |
| **Age (mean (sd))** | 40.7 (13.2) | |
| **Sex (n (%))**  Female  Male | 17 (53.1%)  15 (46.9%) | |
| **Highest level of Education (n (%))**  Below A levels*  A levels or vocational training  Bachelor’s degree and above | 7 (21.9%)  6 (18.8%)  19 (59.4%) | |
| **Ethnicity** **(n (%))**  White  Black  Asian  Mixed | 28 (87.5%)  2 (6.3%)  1 (3.1%)  1 (3.1%) | |

**S3 – Primary analysis – Additional plots**

~~Of the set of pre-specified covariates included in the statistical model, there was a significant main effect of study period with households drinking 358.2ml (95% confidence intervals 229.2 to 487.0, p<0.001) less wine during the second study period compared to the first (Table 3). There was also a significant main effect, with households purchasing larger bottles first, consuming 303.0ml (95% confidence intervals 72.0 to 533.9, p=0.012) less wine per 14-day period compared to households purchasing smaller bottles first (Table 3). There were also significant main effects of baseline consumption, guest consumption, out-of-home consumption and the duration of the ‘usual behaviour’ period. More wine was consumed per 14-day study period when households’ self-reported baseline wine consumption was higher (a 1.93ml increase per 14-day period for each 1ml increase in baseline weekly consumption; 95% confidence intervals 1.80 to 2.06, p<0.001), or reported having guests who drank from their study wine (a 0.66ml increase for each 1ml consumed by guests; 95% confidence intervals 0.42 to 0.91, p<0.001). Less wine was consumed per 14-day study period when households reported drinking wine out of the home (0.14ml decrease for each 1ml consumed out of the home; 95% confidence intervals -0.23 to -0.06, p=0.001). Less wine was also consumed per study 14-day period for each extra log-day spent in the washout period (-378.3ml 95% confidence intervals -249.8.1 to -508.1, p<0.001) (Table 3 main manuscript).~~

**Figure S1:** Wine consumed ((ml) mean) by study Period


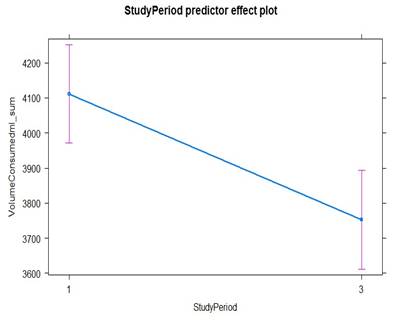


**Figure S2:** Wine consumed ((ml) mean) according bottle size order


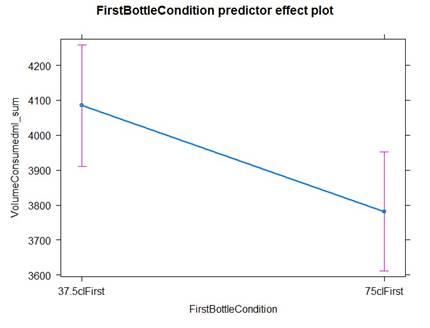


**Table S2:** Mean (*SD*) consumption in ml across households (*n*= 217) with each bottle size (each used for two weeks) and each glass size, according to bottle size order.

|  | | | | | | | **Overall**** | |
| --- | --- | --- | --- | --- | --- | --- | --- | --- |
|  | **75cl** | | | **37.5cl** | | |  |  |
|  | **75cl first** | **37.5cl first** | **Overall*** | **75cl first** | **37.5cl first** | **Overall*** | **75cl first** | **37.5cl first** |
| **290ml glasses** | 3978.4 (1332.1) | 4022.1 (1876.3) | 4000.2 (1619.2) | 3395.7 (1260.9) | 4275.9 (1684.9) | 3835.8 (1545.3) | 3687.0 (1323.3) | 4149.0 (1778.9) |
| **350ml glasses** | 4070.3 (1608.9) | 4252.1 (2918.1) | 4161.2 (2347.6) | 3722.6 (1612.6) | 658.7 (2800.9) | 3835.8 (1545.3) | 3896.4 (1613.1) | 4457.2 (2854.4) |

**S4 – Secondary analysis – Impact of covariates**

There was a significant main effect of bottle size order (0.52 days/1.5l, 95% confidence intervals -0.13 to 0.54), with consumption being slower in households purchasing larger bottles first, compared to households purchasing smaller bottles first (Table 4). There were also significant main effects of baseline consumption, guest consumption and the duration of the ‘usual behaviour’ period. Consumption rate was faster when baseline consumption was higher and if guests drank from households’ study wine. Each extra day spent in the washout period increased the rate of consumption (i.e. slower) (Table S1).

**Table S1:** Mixed-effect regression model, logged estimates and estimates for rate of wine consumption in days/1.5litres (n = 217).

|  |  |  |  | **95%CI for estimate** | |  |
| --- | --- | --- | --- | --- | --- | --- |
|  | **Estimate (SE)** | **t-value** | **P-value** | **Lower** | **Upper** | **Percentage change (95% CIs)** ++ |
| **Intercept** | 5.38 (0.66) | 8.14 | <0.001 | 4.11 | 6.65 | 100 |
| **Bottle size 75cl (ref: 37.5cl)** | -0.16 (0.25) | -0.63 | 0.530 | -0.64 | 0.33 | -2.93 (-11.98, 6.14) |
| **Glass size 350ml (ref: 290ml)** | 0.18 (0.29) | 0.60 | 0.548 | -0.38 | 0.74 | 3.26 (-7.15, 13.68) |
| **Intervention order (ref: 75cl first)** | 0.52 (0.24)* | 2.20 | 0.029 | 0.07 | 0.98 | 9.74 (1.22, 18.26) |
| **Intervention period (ref: period1 )** | 0.20 (0.17) | 1.19 | 0.237 | -0.13 | 0.54 | 3.78 (-2.44, 9.97) |
| **Baseline consumption (ml)** | -0.001 (0.000)** | -7.78 | <0.001 | -0.001 | -0.001 | -0.02 (-0.02, -0.01) |
| **Non-study wine consumption (ml)** | 0.000 (0.001) | 0.44 | 0.660 | -0.001 | 0.001 | 0 (-0.01, 0.02) |
| **Guest consumption (ml)** | -0.001 (0.000)** | -4.05 | <0.001 | -0.002 | -0.001 | -0.02 (-0.03, -0.01) |
| **Out-of-home consumption (ml)** | 0.000 (0.000) | -0.61 | 0.541 | 0.000 | 0.000 | 0 (-0.01, 0) |
| **Number of wine drinkers (ref: 2)** |  |  |  |  |  |  |
| **one** | 0.43 (0.36) | 1.19 | 0.234 | -0.26 | 1.11 | 7.92 (-4.81, 20.67) |
| **three** | 0.66 (0.4) | 1.64 | 0.103 | -0.11 | 1.44 | 12.29 (-2.14, 26.67) |
| **four** | 0.29 (0.87) | 0.33 | 0.740 | -1.38 | 1.96 | 5.37 (-25.65, 36.4) |
| **log(‘Usual behaviour’ period duration (days) + 1)^+^** | 0.58 (0.13)** | 4.35 | <0.001 | 0.32 | 0.84 | 10.78 (6.01, 15.54) |
| **Price (£) per litre** | 0.04 (0.03) | 1.18 | 0.240 | -0.02 | 0.10 | 0.69 (-0.44, 1.82) |
| **Bottle-Glass interaction (ref: 37.5cl & 290ml)** | 0.12 (0.34) | 0.34 | 0.731 | -0.55 | 0.78 | 2.18 (-10.25, 14.45) |

*Significant at the P < 0.05 level; **significant at the P < 0.01 level. CI = confidence interval; SE = standard error.

+Skewed data were transformed. ++Relative to the intercept.

**S5- Sensitivity analyses**

Intention to treat analysis

**Table S1:** Mixed-effect regression model estimates (95% CI) for volume (ml) of wine consumed per 14-day period for ITT sensitivity analysis (n=233)

|  | **Estimate** | **SE** | **t-value** | **p-value** | **95% CI for estimate** | |
| --- | --- | --- | --- | --- | --- | --- |
|  |  |  |  |  | **Lower** | Upper |
| **Intercept** | 1153.2800** | 309.51919 | 3.726 | 0.000225 | 556.2736132 | 1749.652879 |
| **Bottle size 75cl (ref: 37.5cl)** | 180.21118 | 94.73752 | 1.902 | 0.058375 | -3.5226617 | 364.7767883 |
| **Glass size 350ml (ref: 290ml)** | 229.77623 | 134.02701 | 1.714 | 0.087383 | -28.3832331 | 488.2453974 |
| **Intervention order (ref: 75cl first)** | -281.27679** | 117.65712 | -2.391 | 0.017683 | -507.8567273 | -54.71293923 |
| **Intervention period (ref: period 1 )** | -361.29982 | 65.06862 | -5.553 | 7.93E-08 | -487.8157054 | -234.7975734 |
| **Baseline consumption (ml)** | 1.91802** | 0.06715 | 28.564 | <0.000001 | 1.7887157 | 2.04732881 |
| **Non-study wine consumption (ml)** | 0.08249 | 0.22389 | 0.368 | 0.712738 | -0.3504874 | 0.51604158 |
| **Guest consumption (ml)** | 0.66207** | 0.1265 | 5.234 | <0.000001 | 0.4140798 | 0.90569832 |
| **Out-of-home consumption (ml)** | -0.15364** | 0.04278 | -3.591 | 0.000369 | -0.2367762 | -0.07117811 |
| **Number of wine drinkers (ref: 2)** |  |  |  |  |  |  |
| **one** | -74.31435 | 173.73168 | -0.428 | 0.669255 | -408.8624759 | 260.2466838 |
| **three** | -112.98541 | 200.61378 | -0.563 | 0.573856 | -499.2957371 | 273.508046 |
| **four** | -682.79697 | 443.99681 | -1.538 | 0.125557 | -1537.792669 | 172.2024841 |
| **Log (‘Usual behaviour’ period duration (days) + 1)^+^** | -378.0215** | 66.63911 | -5.673 | <0.000001 | -506.3529256 | -249.6965159 |
| **Price (£) per litre** | 1.46906 | 14.23621 | 0.103 | 0.917858 | -25.9769877 | 29.00455022 |
| **Bottle-Glass interaction (ref: 37.5cl & 290ml)** | -191.62387 | 128.97629 | -1.486 | 0.138768 | -443.0569921 | 58.57989274 |

*Significant at the p<0.05 level

** Significant at the p<0.01 level

+Skewed data were transformed.

Conclusions were unchanged with an ITT analysis. This included all households that were randomizedand for which there were primary outcome data available (n= 233) where a mixed model assumes the correlation from complete data cases should be used for the incomplete (with respect to covariates) cases. The difference in consumption of drinking from small bottles compared to large bottles was -180.2ml (95% confidence intervals: -364.8 to 3.52). The main effect of bottle size on wine consumption was not statistically significant (p=0.058). The difference in consumption of drinking from small compared to large was -229.8ml (95% confidence intervals: -488.2 to 28.4). The main effect of glass size on wine consumption was not statistically significant (p=0.087). The difference in consumption when using small bottles with small glasses compared to large bottles with large glasses was -218.4ml (95% confidence intervals: -483.4 to 46.4). The difference between the small bottle-small glass and large bottle-large glass conditions was not significant (p=0.113). The interaction effect was not statistically significant (p=0.139).

Including households violating the protocol

**Table S2:** Mixed-effect regression model estimates (95% CI) for volume (ml) of wine consumed per 14-day period for sensitivity analysis including protocol violators (n=224)

|  | **Estimate** | **SE** | **t-value** | **p-value** | **95% CI for estimate** | |
| --- | --- | --- | --- | --- | --- | --- |
|  |  |  |  |  | **Lower** | Upper |
| **Intercept** | 1145.05223 | 307.06409 | 3.729 | 0.000223 | 553.0117843 | 1736.685721 |
| **Bottle size 75cl (ref: 37.5cl)** | 175.81257 | 94.91817 | 1.852 | 0.065261 | -8.4185525 | 360.5531279 |
| **Glass size 350ml (ref: 290ml)** | 257.14877 | 133.07126 | 1.932 | 0.054155 | 0.8432761 | 513.6900521 |
| **Intervention order (ref: 75cl first)** | -309.68888** | 116.43136 | -2.66 | 0.008413 | -533.859409 | -85.52655151 |
| **Intervention period (ref: period 1 )** | -355.62486 | 65.38231 | -5.439 | 1.405E-07 | -482.6620222 | -228.4213125 |
| **Baseline consumption (ml)** | 1.9178** | 0.06624 | 28.953 | <0.000001 | 1.7902681 | 2.04533117 |
| **Non-study wine consumption (ml)** | 0.04414 | 0.22463 | 0.196 | 0.84433 | -0.390714 | 0.47862521 |
| **Guest consumption (ml)** | 0.65454** | 0.12593 | 5.198 | <0.000001 | 0.4075175 | 0.89702968 |
| **Out-of-home consumption (ml)** | -0.15233** | 0.04262 | -3.574 | 0.000393 | -0.2351143 | -0.07017844 |
| **Number of wine drinkers (ref: 2)** |  |  |  |  |  |  |
| **one** | 5.77357 | 173.6887 | 0.033 | 0.973513 | -328.6299523 | 340.1830229 |
| **three** | -104.44429 | 197.93659 | -0.528 | 0.598251 | -485.5183845 | 276.8262793 |
| **four** | -683.13664 | 437.77324 | -1.56 | 0.120126 | -1525.988894 | 159.7106709 |
| **Log (‘Usual behaviour’ period duration (days) + 1)^+^** | -380.34997** | 65.72746 | -5.787 | <0.000001 | -506.9017217 | -253.8050423 |
| **Price (£) per litre** | 1.8951 | 14.16564 | 0.134 | 0.893638 | -25.4132922 | 29.28129681 |
| **Bottle-Glass interaction (ref: 37.5cl & 290ml)** | -184.61591 | 129.50646 | -1.426 | 0.155413 | -436.9503285 | 66.71881693 |

*Significant at the P < 0.05 level; **significant at the P < 0.01 level. CI = confidence interval; SE = standard error. +Skewed data were transformed.

Conclusions were also unchanged with an analysis with households that completed the study in full, including seven which violated the protocol (n=224). The difference in consumption of drinking from small bottles compared to larger bottles was -175.8ml (95% confidence intervals: -360.6 to 8.4). (Supplement). The main effect of bottle size on wine consumption was not statistically significant (p=0.065). The difference in consumption of drinking from small compared to large was -257.1ml (95% confidence intervals: -513.7 to 0.8). The main effect of glass size on wine consumption was not statistically significant (p=0.054). The difference in consumption when using small bottles with small glasses compared to large bottles with large glasses was -248.3ml (95% confidence intervals: -511.4 to 14.6). This difference was not statistically significant (p=0.070). The interaction effect was not statistically significant (p=0.155).

Awareness of study aims

**Table S3:** Mixed-effect regression model estimates (95% CI) for volume (ml) of wine consumed per 14-day period for sensitivity analysis including variable for awareness of study aims (n=217)

|  | **Estimate** | **SE** | **t-value** | **p-value** | **95% CI for estimate** | |
| --- | --- | --- | --- | --- | --- | --- |
|  |  |  |  |  | **Lower** | Upper |
| **Intercept** | 1082.61763 | 336.75271 | 3.215 | 0.00144 | 436.6577579 | 1729.407095 |
| **Bottle size 75cl (ref: 37.5cl)** | 95.43931 | 99.42484 | 0.96 | 0.33823 | -97.2731462 | 288.8316249 |
| **Glass size 350ml (ref: 290ml)** | 209.2801 | 141.85699 | 1.475 | 0.14123 | -62.6633596 | 481.7407356 |
| **Intervention order (ref: 75cl first)** | -243.59632 | 124.9494 | -1.95 | 0.05273 | -482.9890992 | -4.21200103 |
| **Intervention period (ref: period 1 )** | -392.93935** | 67.43139 | -5.827 | <0.000001 | -523.9192423 | -261.9456406 |
| **Baseline consumption (ml)** | 1.94714** | 0.06969 | 27.941 | <0.000001 | 1.8136279 | 2.08065867 |
| **Non-study wine consumption (ml)** | 0.01884 | 0.24247 | 0.078 | 0.93811 | -0.4487062 | 0.48758103 |
| **Guest consumption (ml)** | 0.7092** | 0.1348 | 5.261 | <0.000001 | 0.4412865 | 0.9677303 |
| **Out-of-home consumption (ml)** | -0.16138** | 0.04451 | -3.626 | 0.00033 | -0.2476168 | -0.07585374 |
| **Number of wine drinkers (ref: 2)** |  |  |  |  |  |  |
| **one** | 1.65726 | 186.03748 | 0.009 | 0.9929 | -354.7780352 | 358.0960522 |
| **three** | -98.43385 | 207.24206 | -0.475 | 0.63533 | -495.3369244 | 299.0817884 |
| **four** | -653.36587 | 443.75912 | -1.472 | 0.14261 | -1503.591111 | 196.8054803 |
| **Log (‘Usual behaviour’ period duration (days) + 1)^+^** | -399.94035** | 68.91881 | -5.803 | <0.000001 | -532.0142846 | -267.9131615 |
| **Price (£) per litre** | 7.3353 | 14.75782 | 0.497 | 0.61944 | -21.0598055 | 35.73440943 |
| **Guessed aim** | -20.47477 | 142.71902 | -0.143 | 0.88608 | -293.9449539 | 252.9289059 |
| **Bottle-Glass interaction (ref: 37.5cl & 290ml)** | -131.26285 | 133.88145 | -0.98 | 0.32809 | -392.2899061 | 127.9950341 |
|  |  |  |  |  |  |  |

*Significant at the P < 0.05 level; **significant at the P < 0.01 level. CI = confidence interval; SE = standard error.

The majority (155/217 (71%) of households correctly guessed the study aim in response to an end-of-study question. The results of the main analysis were unaltered by adding awareness of the study aim as a covariate into the analysis. The difference in consumption of drinking from small bottles compared to large bottles was -95.4ml (95% confidence intervals: -288.8 to 97.3). The main effect of bottle size on wine consumption was not statistically significant (p=0.338). The difference in consumption of drinking from smaller compared to larger glasses was –209.3ml (95% confidence intervals: -481.7 to 62.7). The main effect of glass size on wine consumption was not statistically significant (p=0.141). The interaction effect was not statistically significant (p=0.328). The difference in consumption when using smaller bottles with smaller glasses compared to larger bottles with larger glasses was -173.5ml (95% confidence intervals: -451.4 to 104.3). This difference was not statistically significant (p=0.232).

Mitigating factors affecting consumption

**Table S4:** Mixed-effect regression model estimates (95% CI) for volume (ml) of wine consumed per 14-day period for sensitivity analysis including variable for mitigating factors affecting wine consumption (n=217)

|  | **Estimate** | **SE** | **t-value** | **p-value** | **95% CI for estimate** | |
| --- | --- | --- | --- | --- | --- | --- |
|  |  |  |  |  | **Lower** | Upper |
| **Intercept** | 1049.7507 | 291.17665 | 3.605 | 0.000357 | 488.2028001 | 1609.745407 |
| **Bottle size 75cl (ref: 37.5cl)** | 90.82383 | 89.60092 | 1.014 | 0.311848 | -82.7063028 | 264.8638605 |
| **Glass size 350ml (ref: 290ml)** | 262.62127* | 127.34263 | 2.062 | 0.039999 | 17.6787492 | 507.7405992 |
| **Intervention order (ref: 75cl first)** | -301.35428** | 112.20942 | -2.686 | 0.007832 | -517.157045 | -85.55079144 |
| **Intervention period (ref: period 1 )** | -242.24421** | 62.25014 | -3.891 | 0.000132 | -362.6140466 | -121.0228243 |
| **Baseline consumption (ml)** | 1.89996** | 0.06399 | 29.689 | <0.000001 | 1.7768854 | 2.02303801 |
| **Non-study wine consumption (ml)** | -0.09441 | 0.20846 | -0.453 | 0.650912 | -0.4974519 | 0.307953007 |
| **Guest consumption (ml)** | 0.5374** | 0.11798 | 4.555 | <0.000001 | 0.3063006 | 0.764440083 |
| **Out-of-home consumption (ml)** | -0.0762 | 0.04087 | -1.865 | 0.062976 | -0.1552459 | 0.002541284 |
| **Number of wine drinkers (ref: 2)** |  |  |  |  |  |  |
| **one** | 67.06678 | 167.60344 | 0.4 | 0.689457 | -255.2757124 | 389.4101077 |
| **three** | 26.28691 | 188.76556 | 0.139 | 0.889375 | -336.8449903 | 389.4957249 |
| **four** | -618.47168 | 414.47472 | -1.492 | 0.137183 | -1415.599603 | 178.654459 |
| **Log (‘Usual behaviour’ period duration (days) + 1)^+^** | -327.15507** | 63.02504 | -5.191 | <0.000001 | -448.3668052 | -205.9399143 |
| **Price (£) per litre** | 5.90348 | 13.26783 | 0.445 | 0.65659 | -19.6136749 | 31.62142097 |
| **Mitigating Factors++** | 432.79137** | 51.24011 | 8.446 | <0.000001 | 334.2453506 | 533.2304364 |
| **Bottle-Glass interaction (ref: 37.5cl & 290ml)** | -61.93219 | 120.85382 | -0.512 | 0.608864 | -296.5262645 | 172.4533407 |

*Significant at the P < 0.05 level; **significant at the P < 0.01 level. CI = confidence interval; SE = standard error. +Skewed data were transformed. ++Ranges from -2 to 2 for each fortnight.

A final sensitivity analysis included a variable reflecting whether participants reported any mitigating factors each week (-1 is reduction, 0 is none and +1 is increase) perceived to have affected their consumption during each study period. The difference in consumption of drinking from smaller bottles compared to larger bottles was -90.8ml (95% confidence intervals: -264.9 to 82.7). The main effect of bottle size on wine consumption was not statistically significant (p=0.312). The difference in consumption of drinking from 290ml compared to 350ml was –262.6ml (95% confidence intervals: -507.7 to 17.7). The main effect of glass size on wine consumption was statistically significant (p=0.040). The interaction effect was not statistically significant (p=0.609). The difference in consumption when using smaller bottles with smaller glasses compared to larger bottles with larger glasses was -291.5ml (95% confidence intervals: -543.2 to 40.1). This difference was statistically significant (p=0.026) and the model AIC suggested the mitigating circumstances model was the best fitting compared to all other models on this dataset.

1. **All wines were available in both 75cl and 37.5cl bottles** [↑](#footnote-ref-2)
